# Supplementary material for: Neurological Disorders and Clinical Progression in Boxers from the 20th Century: A Narrative Review
Source: Brain Sci. 2025 Jul 8;15(7):729. doi: 10.3390/brainsci15070729 (PMC12293417; doi:10.3390/brainsci15070729)
Supplement: Supplementary file 1 [file brainsci-15-00729-s001.zip › brainsci-3669367-supplementary.pdf]

brain sciences

Supplementary Materials

Neurological Disorders and Clinical Progression in Boxers from the 20th Century: A Narrative Review

Rudolph J. Castellani [1], Nicolas Kostelecky [2], Jared T. Ahrendsen [1], Malik Nassan [3], Pouya Jamshidi [1], Grant L. Iverson [4-8]

1. Department of Pathology, Northwestern University Feinberg School of Medicine, Chicago, Illinois, USA
2. Department of Pathology, Western Michigan University Homer Styker MD School of Medicine, Kalamazoo, Michigan, USA
3. Mesulam Center for Cognitive Neurology and Alzheimer's Disease; Departments of Neurology and Psychiatry and Behavioral Sciences, Northwestern University Feinberg School of Medicine, Chicago, Illinois, USA
4. Department of Physical Medicine and Rehabilitation, Harvard Medical School, Boston, MA, USA.
5. Department of Physical Medicine and Rehabilitation, Spaulding Rehabilitation Hospital, Charlestown, MA, USA.
6. Department of Physical Medicine and Rehabilitation, Schoen Adams Research Institute at Spaulding Rehabilitation, Charlestown, MA, USA.
7. Home Base, A Red Sox Foundation and Massachusetts General Hospital Program, Charlestown, MA, USA.
8. Mass General for Children Sports Concussion Program, Boston, MA, USA

Table of Contents

Age and Boxing History 2

Frequencies of Neurological Findings: Total Sample 3

Frequencies of Neurological Findings: Stratified by Era of Fighting 6

Raw Data: Sources 10

Additional Content 21

Table S1. Coding disagreements and resolution 21

Table S2. Comparison of articles used in this paper versus Iverson et al. 22

Table S3. Cases in chronological order. 23

Table S4. Cases sorted by progression rating. 28

Table S5. Cases with quotes. 39

Table S6. Boxing history, clinical features, and neuropathology in boxers from Corsellis et al. 1973. 53

Age and Boxing History

Total Sample

		Age	Age_Began_Boxing	Career_Years	Fights
N	Valid	178	80	96	123
	Missing	65	163	147	120
Mean		44.00	14.46	12.9089	197.28
Median		41.00	15.00	11.5000	106.00
Std. Deviation		16.248	2.667	6.45288	223.712
Minimum		13	7	.25	0
Maximum		91	21	32.00	1500
Percentiles	25	29.75	13.00	8.2500	65.00
	50	41.00	15.00	11.5000	106.00
	75	58.00	16.00	18.0000	300.00

By Time Period

Time_Period			Age	Age_Began_Boxing	Career_Years	Fights
After 1973	N	Valid	59	2	27	59
		Missing	15	72	47	15
	Mean		44.78	15.00	10.8611	99.44
	Median		39.00	15.00	10.0000	79.00
	Std. Deviation		16.416	1.414	6.48086	110.587
	Minimum		18	14	.25	0
	Maximum		76	16	25.00	600
	Percentiles	25	31.00	14.00	6.0000	34.00
		50	39.00	15.00	10.0000	79.00
		75	60.00	.	14.0000	115.00
1973 or Before	N	Valid	119	78	69	64
		Missing	50	91	100	105
	Mean		43.61	14.45	13.7101	287.48
	Median		44.00	15.00	12.0000	250.00
	Std. Deviation		16.220	2.695	6.30849	261.584
	Minimum		13	7	2.00	10
	Maximum		91	21	32.00	1500
	Percentiles	25	28.00	13.00	9.0000	100.00
		50	44.00	15.00	12.0000	250.00
		75	57.00	16.00	19.5000	372.50

Frequencies of Neurological Findings: Total Sample

Frequency Tables: Total Sample N=243

Dysarthria

		N	%
No		10	4.1%
Yes		119	49.0%
Missing	System	114	46.9%

Increased reflexes

		N	%
No		16	6.6%
Yes		61	25.1%
Missing	System	166	68.3%

Babinski_sign

		N	%
No		20	8.2%
Yes		38	15.6%
Missing	System	185	76.1%

Abnormal_facies

		N	%
No		2	0.8%
Yes		55	22.6%
Missing	System	186	76.5%

Tremor

		N	%
No		8	3.3%
Yes		46	18.9%
Missing	System	189	77.8%

Intention_ tremor

		N	%
Yes		18	7.4%
Missing	System	225	92.6%

Abnormal_gait

		N	%
No		4	1.6%
Yes		106	43.6%
Missing	System	133	54.7%

Ataxia

		N	%
No		14	5.8%
Yes		54	22.2%
Missing	System	175	72.0%

Bradykinesia_or_slowness_of_movement

		N	%
No		1	0.4%
Yes		30	12.3%
Missing	System	212	87.2%

Visual_loss

		N	%
No		1	0.4%
Yes		14	5.8%
Missing	System	228	93.8%

Abnormal_eye_movements

		N	%
No		7	2.9%
Yes		47	19.3%
Missing	System	189	77.8%

Memory_loss

		N	%
No		10	4.1%
Yes		88	36.2%
Missing	System	145	59.7%

Romberg_sign

		N	%
Yes		13	5.3%
Missing	System	230	94.7%

Pathological_drooling_or_dribbling

		N	%
Yes		10	4.1%
Missing	System	233	95.9%

Headache

		N	%
No		3	1.2%
Yes		44	18.1%
Missing	System	196	80.7%

Cogwheel_rigidity

		N	%
No		2	0.8%
Yes		13	5.3%
Missing	System	228	93.8%

Facial_nerve_paralysis_or_palsy

		N	%
No		4	1.6%
Yes		15	6.2%
Missing	System	224	92.2%

Dysdiadochokinesis

		N	%
No		2	0.8%
Yes		11	4.5%
Missing	System	230	94.7%

Increased_tone_or_spasticity

		N	%
No		6	2.5%
Yes		46	18.9%

Missing	System	191	78.6%

Epilepsy_or_seizures_including_absences

		N	%
Yes		14	5.8%
Missing	System	229	94.2%

Clonus

		N	%
No		1	0.4%
Yes		11	4.5%
Missing	System	231	95.1%

Specific_mention_of_dementia_or_derivative_thereof

		N	%
No		3	1.2%
Yes		42	17.3%
Missing	System	198	81.5%

Frequencies of Neurological Findings: Stratified by Era of Fighting

Dysarthria

Time_Period			N	%
After 1973	Yes		18	24.3%
	Missing	System	56	75.7%
1973 or Before	No		10	5.9%
	Yes		101	59.8%
	Missing	System	58	34.3%

Increased_reflexes

Time_Period			N	%
After 1973	No		1	1.4%
	Yes		8	10.8%
	Missing	System	65	87.8%
1973 or Before	No		15	8.9%
	Yes		53	31.4%
	Missing	System	101	59.8%

Babinski_sign

Time_Period			N	%
After 1973	No		5	6.8%
	Yes		3	4.1%
	Missing	System	66	89.2%
1973 or Before	No		15	8.9%
	Yes		35	20.7%
	Missing	System	119	70.4%

Abnormal_facies

Time_Period			N	%
After 1973	Yes		4	5.4%
	Missing	System	70	94.6%
1973 or Before	No		2	1.2%
	Yes		51	30.2%
	Missing	System	116	68.6%

Tremor

Time_Period			N	%
After 1973	No		4	5.4%
	Yes		5	6.8%
	Missing	System	65	87.8%
1973 or Before	No		4	2.4%
	Yes		41	24.3%
	Missing	System	124	73.4%

Intention_ tremor

Time_Period			N	%
After 1973	Yes		5	6.8%
	Missing	System	69	93.2%
1973 or Before	Yes		13	7.7%
	Missing	System	156	92.3%

Abnormal_gait

Time_Period			N	%
After 1973	No		1	1.4%
	Yes		16	21.6%
	Missing	System	57	77.0%
1973 or Before	No		3	1.8%
	Yes		90	53.3%
	Missing	System	76	45.0%

Ataxia

Time_Period			N	%
After 1973	No		2	2.7%
	Yes		9	12.2%
	Missing	System	63	85.1%
1973 or Before	No		12	7.1%
	Yes		45	26.6%
	Missing	System	112	66.3%

Bradykinesia_or_slowness_of_movement

Time_Period			N	%
After 1973	No		1	1.4%
	Yes		1	1.4%
	Missing	System	72	97.3%
1973 or Before	Yes		29	17.2%
	Missing	System	140	82.8%

Visual_loss

Time_Period			N	%
After 1973	Missing	System	74	100.0%
1973 or Before	No		1	0.6%
	Yes		14	8.3%
	Missing	System	154	91.1%

Abnormal_eye_movements

Time_Period			N	%
After 1973	No		3	4.1%
	Yes		5	6.8%
	Missing	System	66	89.2%
1973 or Before	No		4	2.4%
	Yes		42	24.9%
	Missing	System	123	72.8%

Memory_loss

Time_Period			N	%
After 1973	Yes		13	17.6%
	Missing	System	61	82.4%
1973 or Before	No		10	5.9%
	Yes		75	44.4%
	Missing	System	84	49.7%

Romberg_sign

Time_Period			N	%
After 1973	Yes		4	5.4%
	Missing	System	70	94.6%
1973 or Before	Yes		9	5.3%
	Missing	System	160	94.7%

Pathological_drooling_or_dribbling

Time_Period			N	%
After 1973	Yes		1	1.4%
	Missing	System	73	98.6%
1973 or Before	Yes		9	5.3%
	Missing	System	160	94.7%

Headache

Time_Period			N	%
After 1973	Yes		3	4.1%
	Missing	System	71	95.9%
1973 or Before	No		3	1.8%
	Yes		41	24.3%
	Missing	System	125	74.0%

Cogwheel_rigidity

Time_Period			N	%
After 1973	No		1	1.4%
	Yes		7	9.5%
	Missing	System	66	89.2%
1973 or Before	No		1	0.6%
	Yes		6	3.6%
	Missing	System	162	95.9%

Facial_nerve_paralysis_or_palsy

Time_Period			N	%
After 1973	No		4	5.4%
	Missing	System	70	94.6%
1973 or Before	Yes		15	8.9%
	Missing	System	154	91.1%

Dysdiadochokinesis

Time_Period			N	%
After 1973	No		2	2.7%
	Yes		2	2.7%
	Missing	System	70	94.6%
1973 or Before	Yes		9	5.3%
	Missing	System	160	94.7%

Increased_tone_or_spasticity

Time_Period			N	%
After 1973	No		4	5.4%
	Yes		6	8.1%
	Missing	System	64	86.5%
1973 or Before	No		2	1.2%
	Yes		40	23.7%
	Missing	System	127	75.1%

Epilepsy_or_seizures_including_absences

Time_Period			N	%
After 1973	Yes		1	1.4%
	Missing	System	73	98.6%
1973 or Before	Yes		13	7.7%
	Missing	System	156	92.3%

Clonus

Time_Period			N	%
After 1973	Yes		1	1.4%
	Missing	System	73	98.6%
1973 or Before	No		1	0.6%
	Yes		10	5.9%
	Missing	System	158	93.5%

Specific_mention_of_dementia_or_derivative_thereof

Time_Period			N	%
After 1973	Yes		11	14.9%
	Missing	System	63	85.1%
1973 or Before	No		3	1.8%
	Yes		31	18.3%
	Missing	System	135	79.9%

Raw Data: Sources

	Study ID	Publication Year	Source	Source_Case_ID	Time_Period
1	1	1928	Martland	2	1973 or Before
2	2	1934	Parker	I	1973 or Before
3	3	1934	Parker	II	1973 or Before
4	4	1934	Parker	III	1973 or Before
5	5	1938	Herzog	1	1973 or Before
6	6	1938	Herzog	2	1973 or Before
7	7	1938	Herzog	3	1973 or Before
8	8	1938	Herzog	4	1973 or Before
9	9	1938	Herzog	5	1973 or Before
10	10	1938	Herzog	6	1973 or Before
11	11	1938	Herzog	7	1973 or Before
12	12	1938	Herzog	8	1973 or Before
13	13	1938	Herzog	9	1973 or Before
14	14	1938	Knoll	1	1973 or Before
15	15	1938	Knoll	2	1973 or Before
16	16	1938	Knoll	3	1973 or Before
17	17	1940	Bowman and Blau	7	1973 or Before
18	18	1941	Grewel		1973 or Before
19	19	1944	Bourrat and Micolier	1	1973 or Before
20	20	1944	Bourrat and Micolier	2	1973 or Before
21	21	1949	McAlpine and Page		1973 or Before
22	22	1949	Critchley	A	1973 or Before
23	23	1949	Critchley	B	1973 or Before
24	24	1949	Critchley	C	1973 or Before
25	25	1949	Critchley	D	1973 or Before
26	26	1949	Critchley	E	1973 or Before
27	27	1949	Critchley	F	1973 or Before
28	28	1949	Critchley	G	1973 or Before
29	29	1950	Raevuori-Nalliaa	1	1973 or Before
30	30	1950	Raevuori-Nalliaa	2	1973 or Before
31	31	1953	Schwarz	1	1973 or Before
32	32	1953	Taylor		1973 or Before
33	33	1954	Soeder and Arndt	1	1973 or Before
34	34	1954	Soeder and Arndt	2	1973 or Before
35	35	1954	Soeder and Arndt	3	1973 or Before
36	36	1954	Soeder and Arndt	4	1973 or Before
37	37	1954	Soeder and Arndt	5	1973 or Before
38	38	1954	Brandenburg and Hallervorden		1973 or Before
39	39	1955	Schwarz		1973 or Before
40	40	1956	Bergleiter and Jokl		1973 or Before
41	41	1957	Grahmann and Ule	1	1973 or Before
42	42	1957	Grahmann and Ule	2	1973 or Before
43	43	1957	Grahmann and Ule	3	1973 or Before
44	44	1957	Grahmann and Ule		1973 or Before
45	45	1957	Critchley	2	1973 or Before
46	46	1957	Critchley	3	1973 or Before
47	47	1957	Critchley	4	1973 or Before
48	48	1957	Critchley	8	1973 or Before
49	49	1957	Critchley	9	1973 or Before
50	50	1957	Critchley	10	1973 or Before
51	51	1957	Critchley	11	1973 or Before
52	52	1957	Critchley	12	1973 or Before
53	53	1957	Critchley	13	1973 or Before
54	54	1957	Critchley	14	1973 or Before
55	55	1957	Critchley	15	1973 or Before
56	56	1957	Critchley	16	1973 or Before
57	57	1957	Critchley	17	1973 or Before
58	58	1957	Critchley	18	1973 or Before
59	59	1957	Critchley	19	1973 or Before
60	60	1957	Critchley	20	1973 or Before
61	61	1957	Critchley	21	1973 or Before
62	62	1958	Muller	1	1973 or Before

	Study ID	Publication Year	Source	Source_Case_ID	Time_Period
63	63	1958	Muller	2	1973 or Before
64	64	1958	Muller	3	1973 or Before
65	65	1959	Neubuerger	1	1973 or Before
66	66	1959	Neubuerger	2	1973 or Before
67	67	1960	Wolowska		1973 or Before
68	68	1962	Courville		1973 or Before
69	69	1962	Spillane	1	1973 or Before
70	70	1962	Spillane	2	1973 or Before
71	71	1962	Spillane	3	1973 or Before
72	72	1962	Spillane	4	1973 or Before
73	73	1962	Spillane	5	1973 or Before
74	74	1963	Mawdsley and Ferguson	1	1973 or Before
75	75	1963	Mawdsley and Ferguson	2	1973 or Before
76	76	1963	Mawdsley and Ferguson	3	1973 or Before
77	77	1963	Mawdsley and Ferguson	4	1973 or Before
78	78	1963	Mawdsley and Ferguson	5	1973 or Before
79	79	1963	Mawdsley and Ferguson	6	1973 or Before
80	80	1963	Mawdsley and Ferguson	7	1973 or Before
81	81	1963	Mawdsley and Ferguson	8	1973 or Before
82	82	1963	Mawdsley and Ferguson	9	1973 or Before
83	83	1963	Mawdsley and Ferguson	10	1973 or Before
84	84	1965	Huszar and Kornyey		1973 or Before
85	85	1966	Burger and Marinovjech		1973 or Before
86	86	1967	Goralski and Sypnieewski		1973 or Before
87	87	1968	Constantitidis and Tissot		1973 or Before
88	88	1968	Payne	1	1973 or Before
89	89	1968	Payne	2	1973 or Before
90	90	1968	Payne	3	1973 or Before
91	91	1968	Payne	4	1973 or Before
92	92	1968	Payne	5	1973 or Before
93	93	1968	Payne	6	1973 or Before
94	94	1969	Betti and Ottino		1973 or Before
95	95	1969	Bousseljot	1	1973 or Before
96	96	1969	Bousseljot	2	1973 or Before
97	97	1969	Bousseljot	3	1973 or Before
98	98	1969	Bousseljot	4	1973 or Before
99	99	1969	Bousseljot	5	1973 or Before
100	100	1969	Bousseljot	6	1973 or Before
101	101	1969	Bousseljot	7	1973 or Before
102	102	1969	Bousseljot	8	1973 or Before
103	103	1969	Roberts	1, sample 34	1973 or Before
104	104	1969	Roberts	2, sample 54	1973 or Before
105	105	1969	Roberts	3, sample 19	1973 or Before
106	106	1969	Roberts	4, sample 28	1973 or Before
107	107	1969	Roberts	5, sample 64	1973 or Before
108	108	1969	Roberts	6, sample 58	1973 or Before
109	109	1969	Roberts	7, sample 63	1973 or Before
110	110	1969	Roberts	1 of additional cases similar to 7	1973 or Before
111	111	1969	Roberts	2 of additional cases similar to 7	1973 or Before
112	112	1969	Roberts	3 of additional cases similar to 7	1973 or Before
113	113	1969	Roberts	4 of additional cases similar to 7	1973 or Before
114	114	1969	Roberts	5 of additional cases similar to 7	1973 or Before
115	115	1969	Roberts	6 of additional cases similar to 7	1973 or Before
116	116	1969	Roberts	7 of additional cases similar to 7	1973 or Before
117	117	1969	Roberts	Case 8, sample 23	1973 or Before
118	118	1969	Roberts	1 of additional cases similar to 8	1973 or Before
119	119	1969	Roberts	2 of additional cases similar to 8	1973 or Before
120	120	1969	Roberts	3 of additional cases similar to 8	1973 or Before
121	121	1969	Roberts	4 of additional cases similar to 8	1973 or Before
122	122	1969	Roberts	5 of additional cases similar to 8	1973 or Before
123	123	1969	Roberts	6 of additional cases similar to 8	1973 or Before
124	124	1969	Roberts	7 of additional cases similar to 8	1973 or Before
125	125	1969	Roberts	Case 9, sample 49	1973 or Before
126	126	1969	Roberts	1 of additional cases similar to 9	1973 or Before
127	127	1969	Roberts	2 of additional cases similar to 9	1973 or Before

	Study ID	Publication Year	Source	Source_Case_ID	Time_Period
128	128	1969	Roberts	3 of additional cases similar to 9	1973 or Before
129	129	1969	Roberts	4 of additional cases similar to 9	1973 or Before
130	130	1969	Roberts	5 of additional cases similar to 9	1973 or Before
131	131	1969	Roberts	Case 10, sample 42	1973 or Before
132	132	1969	Roberts	1 of additional cases similar to 10	1973 or Before
133	133	1969	Roberts	2 of additional cases similar to 10	1973 or Before
134	134	1969	Roberts	3 of additional cases similar to 10	1973 or Before
135	135	1969	Roberts	4 of additional cases similar to 10	1973 or Before
136	136	1969	Roberts	Case 11, sample 98	1973 or Before
137	137	1969	Roberts	1 of additional cases similar to 11	1973 or Before
138	138	1969	Roberts	2 of additional cases similar to 11	1973 or Before
139	139	1969	Roberts	3 of additional cases similar to 11	1973 or Before
140	140	1969	Roberts	Unrelated boxing 1, sample 7	1973 or Before
141	141	1969	Roberts	Unrelated boxing 2, sample 79	1973 or Before
142	142	1969	Roberts	Unrelated boxing 3, smaple 36	1973 or Before
143	143	1969	Roberts	Unrelated boxing 4, sample 57	1973 or Before
144	144	1969	Roberts	Unrelated boxing 5, sample 115	1973 or Before
145	145	1969	Roberts	Unrelated boxing 6, sample 174	1973 or Before
146	146	1969	Roberts	Unrelated boxing 7, sample 83	1973 or Before
147	147	1969	Roberts	Unrelated boxing 8, sample 128	1973 or Before
148	148	1969	Roberts	Unrelated boxing 9, sample 101	1973 or Before
149	149	1969	Roberts	Unrelated boxing 10, sample 137	1973 or Before
150	150	1969	Roberts	Unrelated boxing 11, sample 189	1973 or Before
151	151	1969	Johnson	1	1973 or Before
152	152	1969	Johnson	6	1973 or Before
153	153	1969	Johnson	10	1973 or Before
154	154	1969	Johnson	17	1973 or Before
155	155	1973	Corsellis	1	1973 or Before
156	156	1973	Corsellis	2	1973 or Before
157	157	1973	Corsellis	3	1973 or Before
158	158	1973	Corsellis	4	1973 or Before
159	159	1973	Corsellis	5	1973 or Before
160	160	1973	Corsellis	6	1973 or Before
161	161	1973	Corsellis	7	1973 or Before
162	162	1973	Corsellis	8	1973 or Before
163	163	1973	Corsellis	9	1973 or Before
164	164	1973	Corsellis	10	1973 or Before
165	165	1973	Corsellis	11	1973 or Before
166	166	1973	Corsellis	12	1973 or Before
167	167	1973	Corsellis	13	1973 or Before
168	168	1973	Corsellis	14	1973 or Before
169	169	1973	Corsellis	15	1973 or Before
170	170	1974	Harvey		After 1973
171	171	1984	Casson	1	After 1973
172	172	1984	Casson	2	After 1973
173	173	1984	Casson	3	After 1973
174	174	1984	Casson	4	After 1973
175	175	1984	Casson	5	After 1973
176	176	1984	Casson	6	After 1973
177	177	1984	Casson	7	After 1973
178	178	1984	Casson	8	After 1973
179	179	1984	Casson	9	After 1973
180	180	1984	Casson	10	After 1973
181	181	1984	Casson	11	After 1973
182	182	1984	Casson	12	After 1973
183	183	1984	Casson	13	After 1973
184	184	1984	Casson	14	After 1973
185	185	1984	Casson	15	After 1973
186	186	1984	Casson	16	After 1973
187	187	1984	Casson	17	After 1973
188	188	1984	Casson	18	After 1973
189	189	1982	Kaste	1	After 1973
190	190	1982	Kaste	2	After 1973
191	191	1982	Kaste	3	After 1973
192	192	1983	Kaste	4	After 1973

	Study ID	Publication Year	Source	Source_Case_ID	Time_Period
193	193	1984	Kaste	5	After 1973
194	194	1985	Kaste	6	After 1973
195	195	1986	Kaste	7	After 1973
196	196	1987	Kaste	8	After 1973
197	197	1988	Kaste	9	After 1973
198	198	1989	Kaste	10	After 1973
199	199	1990	Kaste	11	After 1973
200	200	1991	Kaste	12	After 1973
201	201	1992	Kaste	13	After 1973
202	202	1993	Kaste	14	After 1973
203	203	1987	Sabharwal	1	After 1973
204	204	1987	Sabharwal	2	After 1973
205	205	1987	Sabharwal	3	After 1973
206	206	1987	Sabharwal	4	After 1973
207	207	1989	Friedman		After 1973
208	208	1992	Hof	2	After 1973
209	209	1992	Hof	3	After 1973
210	210	1995	Jordan		After 1973
211	211	1996	Geddes		After 1973
212	212	1997	Jordan	1	After 1973
213	213	1997	Jordan	2	After 1973
214	214	1997	Jordan	3	After 1973
215	215	1997	Jordan	4	After 1973
216	216	1997	Jordan	5	After 1973
217	217	1997	Jordan	6	After 1973
218	218	1997	Jordan	7	After 1973
219	219	1997	Jordan	8	After 1973
220	220	1997	Jordan	9	After 1973
221	221	1997	Jordan	10	After 1973
222	222	1997	Jordan	11	After 1973
223	223	1997	Jordan	12	After 1973
224	224	1997	Jordan	13	After 1973
225	225	1997	Jordan	14	After 1973
226	226	1997	Jordan	15	After 1973
227	227	1997	Jordan	16	After 1973
228	228	1997	Jordan	17	After 1973
229	229	1997	Jordan	18	After 1973
230	230	1997	Jordan	19	After 1973
231	231	1997	Jordan	20	After 1973
232	232	1997	Jordan	21	After 1973
233	233	1997	Jordan	22	After 1973
234	234	1997	Jordan	23	After 1973
235	235	1997	Jordan	24	After 1973
236	236	1997	Jordan	25	After 1973
237	237	1997	Jordan	26	After 1973
238	238	1997	Jordan	27	After 1973
239	239	1997	Jordan	28	After 1973
240	240	1997	Jordan	29	After 1973
241	241	1997	Jordan	30	After 1973
242	242	1999	Geddes	2	After 1973
243	243	1999	Drachman		After 1973

Raw Data: Selected Neurological Findings

ID	Year	Source	Dysarthria	Abnorm gait	Memory loss	Abnormal reflexes	Abnormal facies	Ataxia	Abnormal Eye movements	Increased tone_or spasticity	Tremor	Headache	Dementia	Babinski sign	Bradykinesia	Intention tremor	Facial_nerve Paralysis or_palsy
1	1928	Martland	Yes	Yes	.	Yes	Yes	.	.	.	Yes	.	No	No	.	.	.
2	1934	Parker	No	Yes	Yes	Yes	No	Yes	Yes	.	Yes	.	.	No	Yes	.	.
3	1934	Parker	Yes	Yes	Yes	Yes	.	.	No	Yes	.	Yes	.	Yes	Yes	.	.
4	1934	Parker	Yes	Yes	Yes	Yes	No	.	No	Yes	No	No	.	Yes	Yes	.	.
5	1938	Herzog	Yes	Yes	Yes	.	.	.	.	.	.	Yes	.	.	.	.	.
6	1938	Herzog	Yes	Yes	.	.	Yes	Yes	.	.	.	.	Yes	.	.	.	.
7	1938	Herzog	.	Yes	.	.	.	Yes	.	.	.	.	.	.	.	.	.
8	1938	Herzog	Yes	Yes	Yes	.	.	Yes	.	.	.	.	.	.	.	.	.
9	1938	Herzog	Yes	Yes	Yes	.	.	Yes	.	.	.	.	Yes	.	.	.	.
10	1938	Herzog	.	Yes	.	.	.	Yes	.	.	.	.	Yes	.	.	.	.
11	1938	Herzog	.	.	Yes	.	.	.	.	.	.	.	.	.	.	.	.
12	1938	Herzog	.	.	Yes	.	.	.	.	.	.	.	.	.	.	.	.
13	1938	Herzog	.	.	.	.	.	.	.	.	.	.	.	.	.	.	.
14	1938	Knoll	Yes	Yes	.	.	.	Yes	.	.	.	.	.	.	.	.	.
15	1938	Knoll	.	.	Yes	.	Yes	.	.	.	.	.	Yes	.	.	.	.
16	1938	Knoll	.	Yes	Yes	.	.	Yes	.	.	.	.	.	.	.	.	.
17	1940	Bowman and Blau	.	.	Yes	.	Yes	.	Yes	.	.	.	.	.	.	.	.
18	1941	Grewel	Yes	Yes	.	Yes	Yes	.	Yes	Yes	Yes	.	.	.	.	.	Yes
19	1944	Bourrat and Micolier	No	.	Yes	.	Yes	.	Yes	.	.	Yes	.	.	.	.	.
20	1944	Bourrat and Micolier	.	.	Yes	.	.	.	.	.	Yes	.	.	.	.	.	.
21	1949	McAlpine and Page	Yes	Yes	.	Yes	Yes	Yes	No	Yes	Yes	.	.	Yes	.	Yes	.
22	1949	Critchley	.	Yes	.	.	.	.	.	.	.	Yes	.	.	.	.	.
23	1949	Critchley	No	.	Yes	No	Yes	.	.	.	.	.	Yes	.	Yes	.	.
24	1949	Critchley	Yes	.	.	.	.	.	Yes	Yes	.	.	Yes	.	.	.	.
25	1949	Critchley	.	.	Yes	.	.	.	.	.	.	Yes	.	.	.	.	.
26	1949	Critchley	Yes	Yes	Yes	No	.	.	Yes	.	No	Yes	.	No	Yes	.	Yes
27	1949	Critchley	.	Yes	Yes	Yes	.	.	Yes	.	No	Yes	.	Yes	Yes	.	.
28	1949	Critchley	.	.	.	.	.	.	Yes	.	.	.	.	.	.	.	.
29	1950	Raevuori-Nalliaa	.	.	Yes	.	.	.	.	.	.	Yes	.	.	.	.	.
30	1950	Raevuori-Nalliaa	Yes	Yes	Yes	Yes	Yes	.	Yes	.	.	Yes	Yes	.	Yes	.	Yes
31	1953	Schwarz	.	Yes	.	.	.	Yes	Yes	.	Yes	Yes	.	Yes	.	Yes	Yes
32	1953	Taylor	Yes	Yes	Yes	No	.	Yes	.	.	.	Yes	.	.	.	.	.
33	1954	Soeder and Arndt	Yes	Yes	.	Yes	Yes	Yes	Yes	.	Yes	.	.	.	.	.	.
34	1954	Soeder and Arndt	.	.	.	Yes	.	.	Yes	.	Yes	Yes	.	.	.	.	Yes
35	1954	Soeder and Arndt	.	.	.	Yes	.	.	Yes	.	.	Yes	.	.	.	.	.
36	1954	Soeder and Arndt	.	.	.	No	.	.	Yes	.	.	Yes	.	.	.	.	Yes
37	1954	Soeder and Arndt	.	Yes	Yes	Yes	.	.	Yes	.	Yes	Yes	.	.	.	.	.
38	1954	Brandenburg and Hallervorden	Yes	Yes	Yes	Yes	.	No	.	Yes	.	.	Yes	Yes	Yes	.	Yes
39	1955	Schwarz	.	.	Yes	.	.	.	.	Yes	.	Yes	.	.	Yes	.	Yes
40	1956	Bergleiter and Jokl	.	Yes	.	Yes	.	Yes	Yes	.	.	.	.	.	.	.	Yes
41	1957	Grahmann and Ule	.	.	Yes	.	.	.	Yes	.	.	Yes	Yes	.	.	.	.
42	1957	Grahmann and Ule	Yes	.	.	.	.	.	.	.	.	.	Yes	.	.	.	.
43	1957	Grahmann and Ule	Yes	Yes	.	Yes	Yes	Yes	.	.	Yes	Yes	.	.	Yes	.	.

ID	Year	Source	Dysarthria	Abnorm gait	Memory loss	Abnormal reflexes	Abnormal facies	Ataxia	Abnormal Eye movements	Increased tone_or spasticity	Tremor	Headache	Dementia	Babinski sign	Bradykinesia	Intention tremor	Facial_nerve Paralysis or_palsy
44	1957	Grahmann and Ule	Yes	Yes	Yes	Yes	Yes	.	.	.	.	.	Yes	.	.	.	.
45	1957	Critchley	Yes	.	.	.	.	.	.	.	.	Yes	.	.	.	.	.
46	1957	Critchley	Yes	.	Yes	.	.	.	.	.	.	.	Yes	.	.	.	.
47	1957	Critchley	.	.	.	.	.	.	.	.	.	.	.	.	.	.	.
48	1957	Critchley	Yes	Yes	Yes	Yes	Yes	Yes	.	.	Yes	.	.	Yes	.	.	.
49	1957	Critchley	Yes	.	.	Yes	.	.	.	.	Yes	.	.	No	.	Yes	.
50	1957	Critchley	Yes	Yes	.	.	.	.	.	.	Yes	.	.	Yes	.	Yes	.
51	1957	Critchley	Yes	Yes	.	.	Yes	.	.	.	Yes	.	.	.	.	Yes	.
52	1957	Critchley	.	.	.	.	.	.	.	.	.	.	.	.	.	.	.
53	1957	Critchley	.	.	.	.	.	.	.	.	.	.	.	.	.	.	.
54	1957	Critchley	Yes	.	Yes	.	.	.	.	.	.	Yes	.	.	.	.	.
55	1957	Critchley	Yes	Yes	Yes	.	.	.	.	.	.	Yes	.	.	.	.	.
56	1957	Critchley	.	.	.	.	.	.	.	.	.	Yes	.	.	.	.	.
57	1957	Critchley	.	Yes	.	.	.	.	.	.	.	.	.	.	.	.	.
58	1957	Critchley	.	.	.	.	.	.	.	.	Yes	.	.	.	.	.	.
59	1957	Critchley	.	.	.	.	.	.	.	.	Yes	.	.	.	.	.	.
60	1957	Critchley	.	.	.	.	.	.	.	.	.	.	.	.	.	.	.
61	1957	Critchley	.	.	.	.	.	.	.	.	.	.	.	.	.	.	.
62	1958	Muller	Yes	Yes	Yes	.	.	.	.	Yes	.	Yes	Yes	.	.	.	.
63	1958	Muller	.	Yes	Yes	.	.	.	.	Yes	.	Yes	.	.	.	.	.
64	1958	Muller	.	.	.	Yes	.	.	Yes	.	.	.	.	Yes	.	.	Yes
65	1959	Neubuerger	.	.	Yes	No	.	.	Yes	Yes	Yes	Yes	.	.	.	.	.
66	1959	Neubuerger	.	Yes	Yes	Yes	.	Yes	.	.	.	.	.	Yes	Yes	.	.
67	1960	Wolowska	.	Yes	Yes	No	.	.	Yes	Yes	.	.	.	Yes	Yes	.	.
68	1962	Courville	.	.	Yes	.	.	.	.	.	.	.	.	.	.	.	.
69	1962	Spillane	Yes	Yes	.	Yes	.	Yes	No	Yes	.	No	No	Yes	.	.	.
70	1962	Spillane	Yes	Yes	Yes	Yes	.	Yes	Yes	.	.	No	Yes	No	Yes	.	.
71	1962	Spillane	No	Yes	Yes	No	.	Yes	Yes	.	Yes	Yes	.	No	.	.	.
72	1962	Spillane	No	.	Yes	.	.	.	.	.	.	Yes	.	.	.	.	.
73	1962	Spillane	Yes	Yes	Yes	Yes	.	.	Yes	.	.	.	No	No	.	.	.
74	1963	Mawdsley and Ferguson	Yes	Yes	Yes	Yes	.	Yes	Yes	.	.	.	Yes	Yes	.	Yes	.
75	1963	Mawdsley and Ferguson	Yes	Yes	Yes	.	Yes	Yes	Yes	Yes	Yes	.	.	No	.	.	.
76	1963	Mawdsley and Ferguson	No	No	Yes	.	.	.	Yes	.	.	Yes	.	Yes	.	.	.
77	1963	Mawdsley and Ferguson	.	.	Yes	.	.	.	Yes	.	.	.	.	.	.	.	.
78	1963	Mawdsley and Ferguson	Yes	Yes	Yes	.	.	Yes	.	Yes	Yes	.	Yes	Yes	.	Yes	.
79	1963	Mawdsley and Ferguson	Yes	Yes	Yes	Yes	Yes	Yes	Yes	Yes	.	.	.	No	.	Yes	.
80	1963	Mawdsley and Ferguson	Yes	Yes	Yes	Yes	.	Yes	Yes	Yes	Yes	.	.	Yes	.	Yes	.
81	1963	Mawdsley and Ferguson	Yes	Yes	Yes	Yes	Yes	Yes	Yes	.	Yes	.	.	Yes	.	Yes	.

ID	Year	Source	Dysarthria	Abnorm gait	Memory loss	Abnormal reflexes	Abnormal facies	Ataxia	Abnormal Eye movements	Increased tone_or spasticity	Tremor	Headache	Dementia	Babinski sign	Bradykinesia	Intention tremor	Facial_nerve Paralysis or_palsy
82	1963	Mawdsley and Ferguson	Yes	.	Yes	.	.	.	.	.	.	Yes	.	.	.	.	.
83	1963	Mawdsley and Ferguson	Yes	.	.	Yes	Yes	.	.	.	.	.	Yes	No	.	.	.
84	1965	Huszar and Kornyey	Yes	.	Yes	Yes	.	.	.	.	Yes	.	.	.	.	Yes	.
85	1966	Burger and Marinovjech	Yes	.	.	.	.	.	.	.	Yes	.	.	.	.	.	.
86	1967	Goralski and Sypnieewski	.	.	.	No	.	.	Yes	Yes	.	Yes	.	.	.	.	Yes
87	1968	Constantitidis and Tissot	Yes	Yes	Yes	.	.	.	.	.	Yes	.	Yes	Yes	.	.	.
88	1968	Payne	.	.	.	.	.	.	.	.	.	.	.	.	.	.	.
89	1968	Payne	Yes	Yes	.	.	.	Yes	.	.	.	Yes	.	.	.	.	.
90	1968	Payne	Yes	.	Yes	.	.	.	.	.	.	Yes	.	.	.	.	.
91	1968	Payne	Yes	Yes	.	.	.	.	.	.	.	Yes	.	.	.	.	.
92	1968	Payne	Yes	Yes	.	.	.	.	.	.	.	.	.	.	.	.	.
93	1968	Payne	.	.	.	.	.	.	.	.	.	.	.	.	.	.	.
94	1969	Betti and Ottino	Yes	Yes	.	.	.	.	.	.	Yes	Yes	.	.	.	.	.
95	1969	Bousseljot	Yes	.	Yes	.	.	.	.	.	.	.	.	Yes	Yes	.	Yes
96	1969	Bousseljot	.	.	.	Yes	.	.	.	.	.	Yes	.	.	.	.	.
97	1969	Bousseljot	.	.	.	.	.	.	.	.	.	.	.	.	.	.	.
98	1969	Bousseljot	.	.	.	No	.	.	.	.	.	Yes	.	.	.	.	.
99	1969	Bousseljot	.	.	.	.	.	.	.	.	.	Yes	.	.	.	.	.
100	1969	Bousseljot	.	.	.	.	.	.	Yes	.	.	Yes	.	.	.	.	Yes
101	1969	Bousseljot	.	.	Yes	.	.	.	.	.	.	Yes	.	.	.	.	.
102	1969	Bousseljot	.	.	.	.	.	.	.	.	.	.	.	.	.	.	.
103	1969	Roberts	Yes	Yes	Yes	Yes	Yes	No	.	Yes	Yes	.	Yes	Yes	Yes	.	.
104	1969	Roberts	Yes	.	No	Yes	Yes	.	Yes	.	Yes	.	.	Yes	.	.	.
105	1969	Roberts	Yes	Yes	Yes	No	.	Yes	.	.	Yes	.	.	Yes	Yes	Yes	.
106	1969	Roberts	Yes	No	Yes	No	Yes	.	.	Yes	Yes	.	.	No	.	.	.
107	1969	Roberts	Yes	Yes	Yes	Yes	Yes	Yes	.	Yes	.	Yes	Yes	Yes	.	.	.
108	1969	Roberts	Yes	Yes	.	Yes	Yes	Yes	Yes	Yes	.	.	Yes	Yes	.	.	.
109	1969	Roberts	Yes	Yes	.	Yes	.	Yes	.	No	.	.	.	No	.	.	.
110	1969	Roberts	Yes	Yes	.	.	Yes	Yes	.	.	.	.	.	.	.	.	.
111	1969	Roberts	Yes	Yes	Yes	.	Yes	Yes	.	.	.	.	.	.	.	.	.
112	1969	Roberts	Yes	Yes	.	.	Yes	Yes	.	.	.	.	.	.	.	.	.
113	1969	Roberts	Yes	Yes	.	.	Yes	Yes	.	.	.	.	.	.	.	.	.
114	1969	Roberts	Yes	Yes	.	.	Yes	Yes	.	.	.	.	.	.	.	.	.
115	1969	Roberts	Yes	Yes	.	.	Yes	Yes	.	.	.	.	.	.	.	.	.
116	1969	Roberts	Yes	Yes	.	.	Yes	Yes	.	.	.	.	.	.	.	.	.
117	1969	Roberts	Yes	Yes	No	Yes	Yes	No	Yes	Yes	.	.	.	No	Yes	.	.
118	1969	Roberts	Yes	Yes	.	Yes	Yes	No	.	Yes	.	.	Yes	.	Yes	.	.
119	1969	Roberts	Yes	Yes	.	Yes	Yes	No	.	Yes	.	.	Yes	.	Yes	.	.
120	1969	Roberts	Yes	Yes	.	Yes	Yes	No	.	Yes	.	.	Yes	.	Yes	.	.
121	1969	Roberts	Yes	Yes	.	Yes	Yes	No	Yes	Yes	.	.	.	.	Yes	.	.
122	1969	Roberts	Yes	Yes	.	Yes	Yes	No	.	Yes	.	.	.	.	Yes	.	.
123	1969	Roberts	Yes	Yes	.	Yes	Yes	No	.	Yes	.	.	.	.	Yes	.	.

ID	Year	Source	Dysarthria	Abnorm gait	Memory loss	Abnormal reflexes	Abnormal facies	Ataxia	Abnormal Eye movements	Increased tone_or spasticity	Tremor	Headache	Dementia	Babinski sign	Bradykinesia	Intention tremor	Facial_nerve Paralysis or_palsy
124	1969	Roberts	Yes	Yes	.	Yes	Yes	No	.	Yes	.	.	.	.	Yes	.	.
125	1969	Roberts	Yes	Yes	Yes	Yes	.	No	.	Yes	.	.	.	Yes	Yes	.	.
126	1969	Roberts	Yes	Yes	.	No	Yes	.	Yes	No	.	.	.	.	.	.	.
127	1969	Roberts	Yes	Yes	.	Yes	Yes	.	.	Yes	.	.	.	.	.	.	.
128	1969	Roberts	Yes	Yes	.	Yes	Yes	.	.	Yes	.	.	.	.	.	.	.
129	1969	Roberts	Yes	Yes	.	Yes	Yes	.	.	Yes	.	.	.	.	.	.	.
130	1969	Roberts	Yes	Yes	.	Yes	Yes	.	.	Yes	.	.	.	.	.	.	.
131	1969	Roberts	Yes	.	.	.	.	.	.	.	.	.	.	Yes	.	.	.
132	1969	Roberts	Yes	Yes	.	.	.	Yes	.	.	.	.	.	Yes	.	.	.
133	1969	Roberts	Yes	.	.	.	.	.	.	.	.	.	.	Yes	.	.	.
134	1969	Roberts	Yes	.	.	.	.	.	.	.	.	.	.	Yes	.	.	.
135	1969	Roberts	Yes	.	.	.	.	.	.	.	.	.	.	Yes	.	.	.
136	1969	Roberts	Yes	.	Yes	.	.	.	.	.	.	.	.	.	.	.	.
137	1969	Roberts	Yes	.	.	.	.	.	.	.	.	.	.	.	.	.	.
138	1969	Roberts	Yes	.	.	.	.	.	.	.	.	.	.	.	.	.	.
139	1969	Roberts	Yes	.	.	.	.	.	.	.	.	.	.	.	.	.	.
140	1969	Roberts	Yes	Yes	No	Yes	.	.	.	Yes	.	.	.	Yes	Yes	.	.
141	1969	Roberts	Yes	Yes	No	Yes	.	.	Yes	Yes	.	.	.	Yes	Yes	.	.
142	1969	Roberts	Yes	Yes	No	No	.	No	Yes	.	Yes	.	.	No	.	.	.
143	1969	Roberts	No	.	Yes	No	Yes	.	.	.	Yes	.	.	Yes	Yes	.	.
144	1969	Roberts	Yes	Yes	No	Yes	.	Yes	Yes	.	.	.	.	Yes	.	.	Yes
145	1969	Roberts	Yes	Yes	No	.	Yes	.	Yes	.	.	.	.	.	.	.	.
146	1969	Roberts	No	Yes	No	Yes	.	.	.	Yes	.	.	.	Yes	.	.	.
147	1969	Roberts	No	.	No	.	Yes	.	.	.	.	.	.	.	.	.	.
148	1969	Roberts	.	.	No	.	.	.	.	.	.	.	.	.	.	.	.
149	1969	Roberts	Yes	.	Yes	.	Yes	.	.	.	.	.	.	.	.	.	.
150	1969	Roberts	.	No	Yes	Yes	.	.	.	Yes	Yes	.	.	No	.	.	.
151	1969	Johnson	Yes	.	Yes	.	.	.	.	.	Yes	.	Yes	.	.	.	.
152	1969	Johnson	.	Yes	Yes	.	.	Yes	.	.	Yes	.	.	.	.	Yes	.
153	1969	Johnson	Yes	.	Yes	.	.	.	.	.	Yes	.	.	.	.	.	.
154	1969	Johnson	Yes	.	Yes	.	.	.	.	.	.	.	.	.	.	.	.
155	1973	Corsellis	Yes	Yes	Yes	.	.	.	.	.	.	.	.	.	.	.	.
156	1973	Corsellis	Yes	Yes	Yes	No	.	Yes	Yes	.	Yes	.	Yes	.	.	.	.
157	1973	Corsellis	Yes	Yes	Yes	.	Yes	Yes	.	Yes	Yes	.	.	.	.	.	.
158	1973	Corsellis	Yes	Yes	Yes	.	Yes	Yes	.	Yes	Yes	.	Yes	.	.	.	.
159	1973	Corsellis	.	.	Yes	.	.	.	.	.	.	.	.	.	.	.	Yes
160	1973	Corsellis	Yes	Yes	Yes	.	Yes	Yes	.	.	.	.	.	.	.	.	.
161	1973	Corsellis	No	Yes	.	.	.	.	.	.	No	Yes	Yes	.	.	.	.
162	1973	Corsellis	.	.	.	.	.	Yes	.	.	Yes	.	.	.	.	.	.
163	1973	Corsellis	Yes	.	.	.	Yes	.	.	.	Yes	.	Yes	.	.	.	.
164	1973	Corsellis	.	.	Yes	.	.	.	.	.	.	.	Yes	.	.	.	.
165	1973	Corsellis	.	.	.	.	.	.	.	.	.	.	.	.	.	.	.
166	1973	Corsellis	.	.	.	.	.	.	.	.	.	.	.	.	.	.	.
167	1973	Corsellis	Yes	.	Yes	.	.	.	.	.	.	.	Yes	.	Yes	.	.
168	1973	Corsellis	.	.	.	.	.	.	.	.	.	.	.	.	.	.	.
169	1973	Corsellis	.	.	.	.	.	.	.	.	.	.	.	.	.	.	.

ID	Year	Source	Dysarthria	Abnorm gait	Memory loss	Abnormal reflexes	Abnormal facies	Ataxia	Abnormal Eye movements	Increased tone_or spasticity	Tremor	Headache	Dementia	Babinski sign	Bradykinesia	Intention tremor	Facial_nerve Paralysis or_palsy
170	1974	Harvey	Yes	Yes	.	Yes	Yes	.	Yes	.	.	.	.	Yes	Yes	.	.
171	1984	Casson	.	.	Yes	.	.	.	.	.	.	.	.	.	.	.	.
172	1984	Casson	.	.	.	.	.	.	.	.	.	.	.	.	.	.	.
173	1984	Casson	.	.	.	.	.	.	.	.	.	.	.	.	.	.	.
174	1984	Casson	.	.	.	.	.	.	.	.	.	.	.	.	.	.	.
175	1984	Casson	.	.	.	.	.	.	.	.	.	.	.	.	.	.	.
176	1984	Casson	.	.	.	.	.	.	.	.	.	.	.	.	.	.	.
177	1984	Casson	Yes	.	.	.	.	.	Yes	.	.	.	.	.	.	.	.
178	1984	Casson	.	.	.	.	.	.	.	.	.	.	.	.	.	.	.
179	1984	Casson	.	.	.	.	.	.	.	.	.	.	.	.	.	.	.
180	1984	Casson	.	.	.	.	.	.	.	.	.	.	.	Yes	.	.	.
181	1984	Casson	.	.	.	.	.	.	.	.	.	.	.	.	.	.	.
182	1984	Casson	.	.	.	.	.	.	.	.	.	.	.	.	.	.	.
183	1984	Casson	.	.	.	.	.	.	.	.	.	.	.	.	.	.	.
184	1984	Casson	.	.	.	.	.	.	.	.	.	.	.	.	.	.	.
185	1984	Casson	.	.	.	.	.	.	.	.	.	.	.	.	.	.	.
186	1984	Casson	.	.	.	.	.	.	.	.	.	.	.	.	.	.	.
187	1984	Casson	.	.	.	.	.	.	.	.	.	.	.	.	.	.	.
188	1984	Casson	.	.	.	.	.	.	.	.	.	.	.	.	.	.	.
189	1982	Kaste	.	Yes	Yes	.	.	.	.	.	.	.	.	.	.	.	.
190	1982	Kaste	.	.	.	.	.	.	.	.	.	.	.	.	.	.	.
191	1982	Kaste	.	.	.	.	.	.	.	.	.	.	.	.	.	.	.
192	1983	Kaste	.	.	.	.	.	.	.	.	.	.	.	.	.	.	.
193	1984	Kaste	.	.	.	.	.	.	.	.	.	.	.	.	.	.	.
194	1985	Kaste	.	.	.	.	.	.	.	.	.	.	.	.	.	.	.
195	1986	Kaste	.	.	.	.	.	.	.	.	.	.	.	.	.	.	.
196	1987	Kaste	.	.	.	.	.	.	.	.	.	.	.	.	.	.	.
197	1988	Kaste	.	.	.	.	.	.	.	.	.	.	.	.	.	.	.
198	1989	Kaste	.	.	.	.	.	.	.	.	.	.	.	.	.	.	.
199	1990	Kaste	.	.	.	.	.	.	.	.	.	.	.	.	.	.	.
200	1991	Kaste	.	.	.	.	.	.	.	.	.	.	.	.	.	.	.
201	1992	Kaste	.	.	.	.	.	.	.	.	.	.	.	.	.	.	.
202	1993	Kaste	.	.	.	.	.	.	.	.	.	.	.	.	.	.	.
203	1987	Sabharwal	Yes	Yes	Yes	Yes	.	Yes	No	No	No	Yes	.	No	.	.	No
204	1987	Sabharwal	Yes	.	Yes	Yes	.	No	Yes	No	.	Yes	.	No	.	.	No
205	1987	Sabharwal	Yes	.	Yes	Yes	.	Yes	No	No	No	.	.	No	.	.	No
206	1987	Sabharwal	Yes	.	Yes	Yes	.	No	Yes	No	No	Yes	.	No	.	.	No
207	1989	Friedman	Yes	Yes	.	Yes	.	Yes	No	Yes	.	.	Yes	Yes	.	.	.
208	1992	Hof	Yes	Yes	Yes	.	Yes	.	.	.	Yes	.	Yes	.	.	.	.
209	1992	Hof	Yes	Yes	Yes	.	Yes	.	.	.	Yes	.	Yes	.	.	.	.
210	1995	Jordan	.	.	.	.	.	.	.	.	.	.	.	.	.	.	.
211	1996	Geddes	.	.	Yes	.	.	.	.	.	.	.	.	.	.	.	.
212	1997	Jordan	.	Yes	.	.	.	Yes	.	Yes	.	.	Yes	.	.	.	.
213	1997	Jordan	.	.	.	.	.	.	.	.	.	.	.	.	.	.	.
214	1997	Jordan	Yes	Yes	.	.	.	.	.	.	.	.	.	.	.	.	.
215	1997	Jordan	.	.	.	.	.	.	.	.	.	.	.	.	.	.	.

ID	Year	Source	Dysarthria	Abnorm gait	Memory loss	Abnormal reflexes	Abnormal facies	Ataxia	Abnormal Eye movements	Increased tone_or spasticity	Tremor	Headache	Dementia	Babinski sign	Bradykinesia	Intention tremor	Facial_nerve Paralysis or_palsy
216	1997	Jordan	.	Yes	.	.	.	.	.	.	.	.	.	.	.	.	.
217	1997	Jordan	.	.	.	.	.	.	.	.	.	.	.	.	.	.	.
218	1997	Jordan	.	.	.	.	.	.	.	.	.	.	.	.	.	.	.
219	1997	Jordan	.	.	.	.	.	.	.	.	.	.	.	.	.	.	.
220	1997	Jordan	.	.	.	.	.	.	.	.	.	.	.	.	.	.	.
221	1997	Jordan	Yes	.	.	.	.	.	.	.	.	.	.	.	.	.	.
222	1997	Jordan	.	.	.	.	.	.	.	.	.	.	.	.	.	.	.
223	1997	Jordan	.	.	.	.	.	.	.	.	.	.	.	.	.	.	.
224	1997	Jordan	.	Yes	.	.	.	.	.	.	.	.	.	.	.	.	.
225	1997	Jordan	.	.	Yes	.	.	.	.	.	.	.	.	.	.	.	.
226	1997	Jordan	Yes	Yes	.	.	.	Yes	.	Yes	.	.	Yes	.	.	Yes	.
227	1997	Jordan	.	.	.	.	.	.	.	.	.	.	.	.	.	.	.
228	1997	Jordan	.	.	.	Yes	.	.	.	.	Yes	.	.	.	.	.	.
229	1997	Jordan	Yes	Yes	Yes	.	.	Yes	.	Yes	.	.	Yes	.	.	.	.
230	1997	Jordan	Yes	Yes	.	.	.	Yes	.	.	.	.	Yes	.	.	Yes	.
231	1997	Jordan	.	.	.	.	.	.	.	.	.	.	.	.	.	.	.
232	1997	Jordan	Yes	Yes	.	Yes	.	Yes	.	Yes	.	.	.	.	.	.	.
233	1997	Jordan	.	.	.	.	.	.	.	.	.	.	.	.	.	.	.
234	1997	Jordan	.	.	.	.	.	.	.	.	Yes	.	.	.	.	.	.
235	1997	Jordan	Yes	.	.	.	.	.	.	.	.	.	.	.	.	.	.
236	1997	Jordan	.	.	.	.	.	.	.	.	.	.	.	.	.	Yes	.
237	1997	Jordan	.	Yes	.	.	.	Yes	Yes	.	.	.	Yes	.	.	Yes	.
238	1997	Jordan	.	.	.	.	.	.	.	.	.	.	.	.	.	.	.
239	1997	Jordan	Yes	Yes	.	.	.	.	.	.	Yes	.	Yes	.	.	Yes	.
240	1997	Jordan	.	.	Yes	.	.	.	.	Yes	.	.	Yes	.	.	.	.
241	1997	Jordan	.	.	.	.	.	.	.	.	.	.	.	.	.	.	.
242	1999	Geddes	.	.	.	.	.	.	.	.	.	.	.	.	.	.	.
243	1999	Drachman	Yes	No	Yes	No	Yes	.	.	.	No	.	Yes	No	No	.	.
243	243	243	129	110	98	77	57	68	54	52	54	47	45	58	31	18	19

A substantial number of cases post-1973 were derived from Kaste et al, Casson et al, and Jordan et al, and many of the neurological findings are coded as not mentioned. This raises the question of whether findings were not mentioned, but present, or not mentioned because they were absent. With regard to this issue:

(i) Kaste et al notes: "Each volunteer underwent detailed neurological examination (by M.K and T.K.) and information was collected on medical history, social variables such as educational and occupational progress compared with siblings and parents, and the boxing career (table I). Boxers were also asked about symptoms connected with fights, especially amnesia and post-concussion symptoms, and about possible sequelae of their boxing careers, such as clumsiness of speech or movements, loss of memory, changes in personality, or other subjective symptoms. They were also asked their views on the importance of their boxing activity to their personal development, education, and occupation.

The psychological examinations (conducted by J. V.) included parts of the Wechsler adult intelligence scale (information, similarities, digit span, block design, and object assembly) as well as associative learning of the Wechsler memory scale. Finnish norms are available for these tests. In addition, we used tests that have been claimed to be sensitive to organic brain damage-the Wisconsin card-sorting test, the trial-making test, the Benton visual retention test, and the Purdue pegboard."

(ii) Casson et al notes: "A detailed medical history was obtained from every subject, with special attention paid to the details of alcohol and other drug use. The boxers underwent the following tests: (1) a noncontrast CT scan of the brain performed with a fourth-generation body scanner, (2) an EEG, (3) a formal neurological examination including mental status, and (4) a battery of neuropsychological tests that included the Trail Making Test, the Digit Symbol Test, the Wechsler Memory Test (including both verbal and visual memory), and the Bender Gestalt Test (the standard administration and a 5-s recall administration). The results of each test were interpreted

independently and without knowledge of the subject's performance on any other examination (CT scan, R.S.; EEG, M.T.; neurological examination, I.C.; and neuropsychological tests, O.S.).

(iii) Jordan et al notes: Boxers underwent a detailed neurologic examination performed by 1 of 3 neurologists (B.DJ, N.R.R, A.RJ). Two examiners were present in 10% of cases to confirm interrater reliability. Impairments were rated using a new scale designed to assess the long-term consequences of brain injury, the Chronic Brain Injury (CBI) scale (Table 1). The CBI scale quantifies the clinical findings of motor, cognitive, and psychiatric deficits associated with boxing-related brain injury as outlined by Méndez: 'Scores on the CBI scale range from 0 to 9, with higher scores reflecting greater impairment. The cognitive component incorporates the boxer's score on the Folstein Mini-Mental State Examination, 26 a widely used cognitive screening instrument. Assessment of behavioral manifestations of CTBI was based on observations during the examination as well as historical information provided by the boxer or the boxer's surrogate responder. The behavioral component of the CBI scale assesses the domains of psychopathology outlined in the Neuropsychiatric Inventory.

From the above, we thought it reasonable to conclude that neurological findings coded as not mentioned were largely or entirely absent in boxers detailed in these three articles.

Additional Content

Table S1. Coding disagreements and resolution

Study ID	Coding disagreement*	Resolution
13	Memory loss	Described as ‘punch drunk’ but memory loss not specifically stated; agreed to code as NM
18	Ataxia	Gait was described as “stomping”; ataxia not specifically noted; agreed to code as NM.
24	Eye movement disorder or other	Eye disorder described as “misty vision (his wife confirmed this)”; eye movement problem not specifically mentioned; agreed to code as NM.
24	Vision loss	Eye disorder described as “misty vision (his wife confirmed this)”; vision loss not specifically mentioned; agreed to code as NM.
40	Abnormal facies	“Facial paresis in the mid and lower branch of the facial nerve”; facial nerve palsies coded separately from abnormal facies; agreed to code as NM.
40	Memory loss	“Decrease in intellectual function developed”; memory loss not specifically mentioned; agreed to code as NM.
64	Abnormal facies	“Weakness of the left facial nerve”; facial nerve palsies coded separately from abnormal facies; agreed to code as NM.
69	“Dementia”	“He was rather slow;” “dementia” not specifically mentioned; agreed to code as NM.
70	“Dementia”	“His memory was unreliable. Psychometric testing revealed moderate intellectual impairment.” “Dementia” not specifically mentioned; agreed to code as NM.
71	Eye movement disorder or other	“His pupils were small but active.” Could not exclude a normal eye examination. Agreed to code as NM.
80	Epilepsy or seizures	“When he was about 50 he lost consciousness for a few minutes.” While seizure is possible, it’s not definitive; agreed to code as NM.
84	Ataxia	“grotesque movements”; “movement of an athetotic nature occurred”; ataxia not specifically mentioned; Agreed to code as NM.
154	Bradykinesia	“On admission he was described as inert and apathetic for long periods,…”; unclear whether this encompassed bradykinesia; agreed to code as NM.
154	Tremor NOS	“On examination, he showed incoordination in the upper limbs”; tremor not specifically noted; agreed to code as NM.
160	Memory loss	The vignette states: “His memory was good”, but later states: “On admission he was orientated but his memory was poor.” Coded as memory loss, objective.

* Coding disagreements between RJC and NAK were identified in 0.28% of the coded entries.

Table S2. Comparison of articles used in this paper versus Iverson et al.

Article include in this review	# of Cases	Articles from Iverson et al.	# of Cases
Martland (1928)	1	Martland (1928)	1
Parker (1934)	3	Parker (1934)	3
Herzog (1938)*	9	(Not included)	
Knoll (1938)*	3	(Not included)	
Bowman and Blau (1940)	1	(Not included)	
Grewel (1941)*	1	(Not included)	
Bourrat and Micolier (1944)*	2	(Not included)	
McAlpine (1949)	1	(Not included)	
Critchley (1949)	7	Critchley (1949)	7
Raevuori-Nallinmaa (1950)	2	Raevuori-Nallinmaa (1950)	2
Schwarz (1953)*	1	(Not included)	
Taylor (1953)*	1	(Not included)	
Soeder and Arndt (1954)*	5	(Not included)	
Brandenburg and Hallervorden (1954)*	1	(Not included)	
Schwarz (1955)*	1	(Not included)	
Bergleiter and Jokl (1956)*	1	(Not included)	
Grahmann and Ule (1957)**	4	(Not included)	
Critchley (1957)	17	Critchley (1957)	7
Muller (1958)	3	(Not included)	
Neubuerger et al. (1959)	2	Neubuerger et al. (1959)	2
Wolowska (1960)*	1	(Not included)	
Courville (1962)	1	Courville (1962)	
Spillane (1962)	5	Spillane (1962)	
Mawdsley and Ferguson (1963)	10	Mawdsley and Ferguson (1963)	10
Huszar and Kornyey (1965)*	1	(Not included)	
Burger and Marinovjech (1966)*	1	(Not included)	
Goralski and Sypniewski (1967)*	1	(Not included)	
Constantitidis and Tissot (1968)***	1	(Not included)	
Payne (1968)	6	Payne (1968)	6
Betti and Ottino (1969)	1	(Not included)	
Bousseljot (1969)	8	(Not included)	
Roberts (1969)[#]	48	Roberts (1969)	11
Johnson (1969)[##]	4	Johnson (1969)	15
Corsellis et al. (1973)	15	Corsellis et al. (1973)	15
Harvey and Davis (1974)	1	Harvey and Davis (1974)	1
Kaste et al. (1982)	14	Kaste et al. (1982)	14
Casson et al. (1984)	18	Casson et al. (1984)	18
Sabharwal et al. (1987)	4	(Not included)	
Friedman (1989)	1	(Not included)	
Hof et al. (1992)[###]	2	Hof et al. (1992)	3
Jordan et al. (1995)	1	Jordan et al. (1995)	1
Geddes et al. (1996)	1	(Not included)	
Jordan et al. (1997)	30	Jordan et al. (1997)	30
Geddes et al. (1999)[@]	1	Geddes et al. (1999)	4
(no author listed) (1999)	1	(no author listed) (1999)	1

* Partially translated in Boxing: Medical Aspects. ** Partially translated in Boxing: Medical Aspects; one case translated with Google Translate. *** Translated with Google Translate. [#] See manuscript, indicating the reason for including 48 cases in the current paper versus Iverson et al. [##]Current paper included only those cases detailed in paragraph form. [###]Iverson et al included case 3, which is the same boxer described in Constantinidis and Tissot (1968).
[@]One case in Iverson et al also reported in Geddes et al. (1996); Iverson et al. includes three non-boxers in their supplement but these were not included in their statistical analyses.

Iverson, G. L., Kissinger-Knox, A., Huebschmann, N. A., Castellani, R. J., & Gardner, A. J. (2023). A narrative review of psychiatric features of traumatic encephalopathy syndrome as conceptualized in the 20th century. Frontiers in neurology, 14, 1214814. https://doi.org/10.3389/fneur.2023.1214814

Table S3. Cases in chronological order.

Author, case	Progression Rating
Martland (1928), case 2	NDD-like
Parker (1934), Case 1	Improvement; motor
Parker (1934), Case 2	Improvement; motor
Parker (1934), Case 3	Improvement; motor
Herzog (1938) Case 1	NM
Herzog (1938) Case 2	NM
Herzog (1938) Case 3	NM
Herzog (1938) Case 4	NM
Herzog (1938) Case 5	NM
Herzog (1938) Case 6	NM
Herzog (1938) Case 7	NM
Herzog (1938) Case 8	NM
Herzog (1938) Case 9	NM
Knoll (1938) Case 1	NM
Knoll (1938) Case 2	NM
Knoll (1938) Case 3	NM
Bowman and Blau (1940)	Stationary
Grewel (1941)	Motor
Bourrat and Micolier (1944), case 1	Stationary
Bourrat and Micolier (1944), case 2	NM
McAlpine and Page (1949)	Motor
Critchley (1949) Case A	NM
Critchley (1949) Case B	NM
Critchley (1949) Case C	NM
Critchley (1949) Case D	NM
Critchley (1949) Case E	NM
Critchley (1949) Case F	NM
Critchley (1949) Case G	NM
Raevuori-Nallinmaa (1950), case 1	Stationary, improvement
Raevuori-Nallinmaa (1950), case 2	Motor and memory
Schwarz (1953)	NM
Taylor (1953)	Improvement
Soeder and Arndt (1954), Case 1	NM
Soeder and Arndt (1954), Case 2	NM
Soeder and Arndt (1954), Case 3	NM
Soeder and Arndt (1954), Case 4	NM
Soeder and Arndt (1954), Case 5	NM
Brandenburg and Hallervorden (1954)	NDD-like
Schwarz (1955)	NM
Bergleiter and Jokl (1956)	Complex
Grahmann and Ule (1957), case 1	Complex
Grahmann and Ule (1957), case 2	Complex
Grahmann and Ule (1957), case 3	Complex
Grahmann and Ule (1957), case with pathology	NDD-like
Critchley (1957), case 2	Complex
Critchley (1957), case 3	NM
Critchley (1957), case 4	NM
Critchley (1957), case 8	Stationary, motor
Critchley (1957), case 9	Motor
Critchley (1957), case 10	Motor
Critchley (1957), case 11	Motor
Critchley (1957), Case 12	NM
Critchley (1957), Case 13	NM
Critchley (1957), Case 14	NM

Author, case	Progression Rating
Critchley (1957), Case 15	NM
Critchley (1957), Case 16	NM
Critchley (1957), Case 17	NM
Critchley (1957), case 18	Improvement
Critchley (1957), case 19	Complex
Critchley (1957), Case 20	NM
Critchley (1957), Case 21	NM
Muller (1958), case 1	Complex
Muller (1958), case 2	NM
Muller (1958), case 3	Complex
Neubuerger (1959), case 1	NM
Neubuerger (1959), case 2	NDD-like
Wolowski (1960)	Motor
Courville (1962)	NM
Spillane (1962), case 1	Motor
Spillane (1962), case 2	Motor, complex
Spillane (1962), case 3	NDD-like
Spillane (1962), case 4	NM
Spillane (1962), case 5	NM
Mawdsley and Ferguson (1963), case 1 (? Johnson case 1)	Motor and memory
Mawdsley and Ferguson (1963), case 2	Motor
Mawdsley and Ferguson (1963), case 3	Memory only
Mawdsley and Ferguson (1963), case 4	Memory only
Mawdsley and Ferguson (1963), case 5	Motor
Mawdsley and Ferguson (1963), case 6	Motor
Mawdsley and Ferguson (1963), case 7	Motor and memory
Mawdsley and Ferguson (1963), case 8	Complex
Mawdsley and Ferguson (1963), case 9	NM
Mawdsley and Ferguson (1963), case 10	Memory only, complex
Huszar and Kornvey (1965)	Motor
Burger and Marinoviech	NM
Goralski and Sypniewski (1967)	Improvement, complex
Constantinidis and Tissot (1968)	Complex
Payne (1968), case 1	NM
Payne (1968), case 2	NM
Payne (1968), case 3	NM
Payne (1968), case 4	Stationary
Payne (1968), case 5	NM
Payne (1968), case 6	NM
Betti and Ottino (1969)	NM
Bousseljot (1969), Case 1	Stationary
Bousseljot (1969), Case 2	NM
Bousseljot (1969), Case 3	NM
Bousseljot (1969), Case 4	NM
Bousseljot (1969), Case 5	NM
Bousseljot (1969), Case 6	NM
Bousseljot (1969), Case 7	NM
Bousseljot (1969), Case 8	NM
Roberts (1969), Case 1	Stationary, motor
Roberts (1969), Case 2	Motor
Roberts (1969), Case 3	Motor
Roberts (1969), Case 4	Memory only
Roberts (1969), Case 5	NM
Roberts (1969), Case 6	Motor and Memory
Roberts (1969), Case 7	Complex
Roberts (1969) Case 1 similar to case 7	Stationary

Author, case	Progression Rating
Roberts (1969) Case 2 similar to case 7	Stationary
Roberts (1969) Case 3 similar to case 7	Stationary
Roberts (1969) Case 4 similar to case 7	Stationary
Roberts (1969) Case 5 similar to case 7	Stationary
Roberts (1969) Case 6 similar to case 7	Stationary
Case 7 similar to case 7	Stationary
Roberts (1969), Case 8	Stationary
Roberts (1969), Case 1 similar to case 8	NM
Roberts (1969), Case 2 similar to case 8	NM
Roberts (1969), Case 3 similar to case 8	NM
Roberts (1969), Case 4 similar to case 8	NM
Roberts (1969), Case 5 similar to case 8	NM
Roberts (1969), Case 6 similar to case 8	NM
Roberts (1969), Case 7 similar to case 8	NM
Roberts (1969), Case 9	Motor
Roberts (1969), Case 1 similar to case 9	NM
Roberts (1969), Case 2 similar to case 9	NM
Roberts (1969), Case 3 similar to case 9	NM
Roberts (1969), Case 4 similar to case 9	NM
Roberts (1969), Case 5 similar to case 9	NM
Roberts (1969), Case 10	Stationary
Roberts (1969), Case 1 similar to case 10	NM
Roberts (1969), Case 2 similar to case 10	NM
Roberts (1969), Case 3 similar to case 10	NM
Roberts (1969), Case 4 similar to case 10	NM
Roberts (1969), Case 11	NM
Roberts (1969), Case 1 similar to case 11	NM
Roberts (1969), Case 2 similar to case 11	NM
Roberts (1969), Case 3 similar to case 11	NM
Roberts (1969), Case 1 unrelated to boxing	NM
Roberts (1969), Case 2 unrelated to boxing	Motor and memory
Roberts (1969), Case 3 unrelated to boxing	NM
Roberts (1969), Case 4 unrelated to boxing	NDD-like
Roberts (1969), Case 5 unrelated to boxing	Improvement
Roberts (1969), Case 6 unrelated to boxing	NM
Roberts (1969), Case 7 unrelated to boxing	NM
Roberts (1969), Case 8 unrelated to boxing	NM
Roberts (1969), Case 9 unrelated to boxing	NM
Roberts (1969), Case 10 unrelated to boxing	Memory only
Roberts (1969), Case 11 unrelated to boxing	NM
Johnson (1969), case 1	Complex
Johnson (1969), case 6	Stationary, motor and memory
Johnson (1969), case 10	Stationary
Johnson (1969), case 17	Complex
Corsellis (1973), Case 1	Complex
Corsellis (1973), Case 2	Complex
Corsellis (1973), Case 3	Complex
Corsellis (1973), Case 4	NDD-like
Corsellis (1973), Case 5	Complex
Corsellis (1973), Case 6	Complex
Corsellis (1973), Case 7	Complex
Corsellis (1973), Case 8	6
Corsellis (1973), Case 9	Complex
Corsellis (1973), Case 10	NDD-like
Corsellis (1973), Case 11	Stationary
Corsellis (1973), Case 12	NM

Author, case	Progression Rating
Corsellis (1973), Case 13	NDD-like
Corsellis (1973), Case 14	NM
Corsellis (1973), Case 15	NM
Harvey (1974)	Motor
Kaste et al. (1982), Case 1	NM
Kaste et al. (1982), Case 2	NM
Kaste et al. (1982), Case 3	NM
Kaste et al. (1982), Case 4	NM
Kaste et al. (1982), Case 5	NM
Kaste et al. (1982), Case 6	NM
Kaste et al. (1982), Case 7	NM
Kaste et al. (1982), Case 8	NM
Kaste et al. (1982), Case 9	NM
Kaste et al. (1982), Case 10	NM
Kaste et al. (1982), Case 11	NM
Kaste et al. (1982), Case 12	NM
Kaste et al. (1982), Case 13	NM
Kaste et al. (1982), Case 14	NM
Casson et al. (1984), Case 1	NM
Casson et al. (1984), Case 2	NM
Casson et al. (1984), Case 3	NM
Casson et al. (1984), Case 4	NM
Casson et al. (1984), Case 5	NM
Casson et al. (1984), Case 6	NM
Casson et al. (1984), Case 7	NM
Casson et al. (1984), Case 8	NM
Casson et al. (1984), Case 9	NM
Casson et al. (1984), Case 10	NM
Casson et al. (1984), Case 11	NM
Casson et al. (1984), Case 12	NM
Casson et al. (1984), Case 13	NM
Casson et al. (1984), Case 14	NM
Casson et al. (1984), Case 15	NM
Casson et al. (1984), Case 16	NM
Casson et al. (1984), Case 17	NM
Casson et al. (1984), Case 18	NM
Sabharwal et al. (1987), Case 1	NM
Sabharwal et al. (1987), Case 2	NM
Sabharwal et al. (1987), Case 3	NM
Sabharwal et al. (1987), Case 4	NM
Friedman (1989)	NDD-like
Hof et al. (1992), case 1	NM
Hof et al. (1992), case 2	NM
Jordan et al. (1995)	NDD-like
Geddes et al. (1996)	NM
Jordan et al. (1997), case 1	NDD-like
Jordan et al. (1997), case 2	NM
Jordan et al. (1997), case 3	NM
Jordan et al. (1997), case 4	NM
Jordan et al. (1997), case 5	NM
Jordan et al. (1997), case 6	NM
Jordan et al. (1997), case 7	NM
Jordan et al. (1997), case 8	NM
Jordan et al. (1997), case 9	NM
Jordan et al. (1997), case 10	NM
Jordan et al. (1997), case 11	NM

Author, case	Progression Rating
Jordan et al. (1997), case 12	NM
Jordan et al. (1997), case 13	NM
Jordan et al. (1997), case 14	NM
Jordan et al. (1997), case 15	NM
Jordan et al. (1997), case 16	NM
Jordan et al. (1997), case 17	NM
Jordan et al. (1997), case 18	NM
Jordan et al. (1997), case 19	NM
Jordan et al. (1997), case 20	NM
Jordan et al. (1997), case 21	NM
Jordan et al. (1997), case 22	NM
Jordan et al. (1997), case 23	NM
Jordan et al. (1997), case 24	NM
Jordan et al. (1997), case 25	NM
Jordan et al. (1997), case 26	NM
Jordan et al. (1997), case 27	NM
Jordan et al. (1997), case 28	NM
Jordan et al. (1997), case 29	NM
Jordan et al. (1997), case 30	NM
Geddes et al. (1999)	NM
Drachman (1999)	NDD-like

Table S4. Cases sorted by progression rating.

Author, case	Progression Rating
Corsellis (1973), Case 8	Complex
Bergleiter and Jokl (1956)	Complex
Grahmann and Ule (1957), case 1	Complex
Grahmann and Ule (1957), case 2	Complex
Grahmann and Ule (1957), case 3	Complex
Critchley (1957), case 2	Complex
Critchley (1957), case 19	Complex
Muller (1958), case 1	Complex
Muller (1958), case 3	Complex
Mawdsley and Ferguson (1963), case 8	Complex
Constantinidis and Tissot (1968)	Complex
Roberts (1969), Case 7	Complex
Johnson (1969), case 1	Complex
Johnson (1969), case 17	Complex
Corsellis (1973), Case 1	Complex
Corsellis (1973), Case 2	Complex
Corsellis (1973), Case 3	Complex
Corsellis (1973), Case 5	Complex
Corsellis (1973), Case 6	Complex
Corsellis (1973), Case 7	Complex
Corsellis (1973), Case 9	Complex
Taylor (1953)	Improvement
Critchley (1957), case 18	Improvement
Roberts (1969), Case 5 unrelated to boxing	Improvement
Goralski and Sypniewski (1967)	Improvement, complex
Parker (1934), Case 1	Improvement; motor
Parker (1934), Case 2	Improvement; motor
Parker (1934), Case 3	Improvement; motor
Mawdsley and Ferguson (1963), case 3	Memory only
Mawdsley and Ferguson (1963), case 4	Memory only
Roberts (1969), Case 4	Memory only
Roberts (1969), Case 10 unrelated to boxing	Memory only
Mawdsley and Ferguson (1963), case 10	Memory only, complex
Grewel (1941)	Motor
McAlpine and Page (1949)	Motor
Critchley (1957), case 9	Motor
Critchley (1957), case 10	Motor
Critchley (1957), case 11	Motor
Wolowski (1960)	Motor
Spillane (1962), case 1	Motor
Mawdsley and Ferguson (1963), case 2	Motor
Mawdsley and Ferguson (1963), case 5	Motor
Mawdsley and Ferguson (1963), case 6	Motor
Huszar and Kornvey (1965)	Motor
Roberts (1969), Case 2	Motor
Roberts (1969), Case 3	Motor
Roberts (1969), Case 9	Motor
Harvey (1974)	Motor
Raevuori-Nallinmaa (1950), case 2	Motor and memory
Mawdsley and Ferguson (1963), case 1 (? Johnson case 1)	Motor and memory
Mawdsley and Ferguson (1963), case 7	Motor and memory
Roberts (1969), Case 6	Motor and Memory
Roberts (1969), Case 2 unrelated to boxing	Motor and memory
Spillane (1962), case 2	Motor, complex

Author, case	Progression Rating
Martland (1928), case 2	NDD-like
Brandenburg and Hallervorden (1954)	NDD-like
Grahmann and Ule (1957), case with pathology	NDD-like
Neubuerger (1959), case 2	NDD-like
Spillane (1962), case 3	NDD-like
Roberts (1969), Case 4 unrelated to boxing	NDD-like
Corsellis (1973), Case 4	NDD-like
Corsellis (1973), Case 10	NDD-like
Corsellis (1973), Case 13	NDD-like
Friedman (1989)	NDD-like
Jordan et al. (1995)	NDD-like
Jordan et al. (1997), case 1	NDD-like
Drachman (1999)	NDD-like
Herzog (1938) Case 1	NM
Herzog (1938) Case 2	NM
Herzog (1938) Case 3	NM
Herzog (1938) Case 4	NM
Herzog (1938) Case 5	NM
Herzog (1938) Case 6	NM
Herzog (1938) Case 7	NM
Herzog (1938) Case 8	NM
Herzog (1938) Case 9	NM
Knoll (1938) Case 1	NM
Knoll (1938) Case 2	NM
Knoll (1938) Case 3	NM
Bourrat and Micolier (1944), case 2	NM
Critchley (1949) Case A	NM
Critchley (1949) Case B	NM
Critchley (1949) Case C	NM
Critchley (1949) Case D	NM
Critchley (1949) Case E	NM
Critchley (1949) Case F	NM
Critchley (1949) Case G	NM
Schwarz (1953)	NM
Soeder and Arndt (1954), Case 1	NM
Soeder and Arndt (1954), Case 2	NM
Soeder and Arndt (1954), Case 3	NM
Soeder and Arndt (1954), Case 4	NM
Soeder and Arndt (1954), Case 5	NM
Schwarz (1955)	NM
Critchley (1957), case 3	NM
Critchley (1957), case 4	NM
Critchley (1957), Case 12	NM
Critchley (1957), Case 13	NM
Critchley (1957), Case 14	NM
Critchley (1957), Case 15	NM
Critchley (1957), Case 16	NM
Critchley (1957), Case 17	NM
Critchley (1957), Case 20	NM
Critchley (1957), Case 21	NM
Muller (1958), case 2	NM
Neubuerger (1959), case 1	NM
Courville (1962)	NM
Spillane (1962), case 4	NM
Spillane (1962), case 5	NM
Mawdsley and Ferguson (1963), case 9	NM

Author, case	Progression Rating
Burger and Marinoviech	NM
Payne (1968), case 1	NM
Payne (1968), case 2	NM
Payne (1968), case 3	NM
Payne (1968), case 5	NM
Payne (1968), case 6	NM
Betti and Ottino (1969)	NM
Bousseljot (1969), Case 2	NM
Bousseljot (1969), Case 3	NM
Bousseljot (1969), Case 4	NM
Bousseljot (1969), Case 5	NM
Bousseljot (1969), Case 6	NM
Bousseljot (1969), Case 7	NM
Bousseljot (1969), Case 8	NM
Roberts (1969), Case 5	NM
Roberts (1969), Case 1 similar to case 8	NM
Roberts (1969), Case 2 similar to case 8	NM
Roberts (1969), Case 3 similar to case 8	NM
Roberts (1969), Case 4 similar to case 8	NM
Roberts (1969), Case 5 similar to case 8	NM
Roberts (1969), Case 6 similar to case 8	NM
Roberts (1969), Case 7 similar to case 8	NM
Roberts (1969), Case 1 similar to case 9	NM
Roberts (1969), Case 2 similar to case 9	NM
Roberts (1969), Case 3 similar to case 9	NM
Roberts (1969), Case 4 similar to case 9	NM
Roberts (1969), Case 5 similar to case 9	NM
Roberts (1969), Case 1 similar to case 10	NM
Roberts (1969), Case 2 similar to case 10	NM
Roberts (1969), Case 3 similar to case 10	NM
Roberts (1969), Case 4 similar to case 10	NM
Roberts (1969), Case 11	NM
Roberts (1969), Case 1 similar to case 11	NM
Roberts (1969), Case 2 similar to case 11	NM
Roberts (1969), Case 3 similar to case 11	NM
Roberts (1969), Case 1 unrelated to boxing	NM
Roberts (1969), Case 3 unrelated to boxing	NM
Roberts (1969), Case 6 unrelated to boxing	NM
Roberts (1969), Case 7 unrelated to boxing	NM
Roberts (1969), Case 8 unrelated to boxing	NM
Roberts (1969), Case 9 unrelated to boxing	NM
Roberts (1969), Case 11 unrelated to boxing	NM
Corsellis (1973), Case 12	NM
Corsellis (1973), Case 14	NM
Corsellis (1973), Case 15	NM
Kaste et al. (1982), Case 1	NM
Kaste et al. (1982), Case 2	NM
Kaste et al. (1982), Case 3	NM
Kaste et al. (1982), Case 4	NM
Kaste et al. (1982), Case 5	NM
Kaste et al. (1982), Case 6	NM
Kaste et al. (1982), Case 7	NM
Kaste et al. (1982), Case 8	NM
Kaste et al. (1982), Case 9	NM
Kaste et al. (1982), Case 10	NM
Kaste et al. (1982), Case 11	NM

Author, case	Progression Rating
Kaste et al. (1982), Case 12	NM
Kaste et al. (1982), Case 13	NM
Kaste et al. (1982), Case 14	NM
Casson et al. (1984), Case 1	NM
Casson et al. (1984), Case 2	NM
Casson et al. (1984), Case 3	NM
Casson et al. (1984), Case 4	NM
Casson et al. (1984), Case 5	NM
Casson et al. (1984), Case 6	NM
Casson et al. (1984), Case 7	NM
Casson et al. (1984), Case 8	NM
Casson et al. (1984), Case 9	NM
Casson et al. (1984), Case 10	NM
Casson et al. (1984), Case 11	NM
Casson et al. (1984), Case 12	NM
Casson et al. (1984), Case 13	NM
Casson et al. (1984), Case 14	NM
Casson et al. (1984), Case 15	NM
Casson et al. (1984), Case 16	NM
Casson et al. (1984), Case 17	NM
Casson et al. (1984), Case 18	NM
Sabharwal et al. (1987), Case 1	NM
Sabharwal et al. (1987), Case 2	NM
Sabharwal et al. (1987), Case 3	NM
Sabharwal et al. (1987), Case 4	NM
Hof et al. (1992), case 1	NM
Hof et al. (1992), case 2	NM
Geddes et al. (1996)	NM
Jordan et al. (1997), case 2	NM
Jordan et al. (1997), case 3	NM
Jordan et al. (1997), case 4	NM
Jordan et al. (1997), case 5	NM
Jordan et al. (1997), case 6	NM
Jordan et al. (1997), case 7	NM
Jordan et al. (1997), case 8	NM
Jordan et al. (1997), case 9	NM
Jordan et al. (1997), case 10	NM
Jordan et al. (1997), case 11	NM
Jordan et al. (1997), case 12	NM
Jordan et al. (1997), case 13	NM
Jordan et al. (1997), case 14	NM
Jordan et al. (1997), case 15	NM
Jordan et al. (1997), case 16	NM
Jordan et al. (1997), case 17	NM
Jordan et al. (1997), case 18	NM
Jordan et al. (1997), case 19	NM
Jordan et al. (1997), case 20	NM
Jordan et al. (1997), case 21	NM
Jordan et al. (1997), case 22	NM
Jordan et al. (1997), case 23	NM
Jordan et al. (1997), case 24	NM
Jordan et al. (1997), case 25	NM
Jordan et al. (1997), case 26	NM
Jordan et al. (1997), case 27	NM
Jordan et al. (1997), case 28	NM
Jordan et al. (1997), case 29	NM

Author, case	Progression Rating
Jordan et al. (1997), case 30	NM
Geddes et al. (1999)	NM
Bowman and Blau (1940)	Stationary
Bourrat and Micolier (1944), case 1	Stationary
Payne (1968), case 4	Stationary
Bousseljot (1969), Case 1	Stationary
Roberts (1969) Case 1 similar to case 7	Stationary
Roberts (1969) Case 2 similar to case 7	Stationary
Roberts (1969) Case 3 similar to case 7	Stationary
Roberts (1969) Case 4 similar to case 7	Stationary
Roberts (1969) Case 5 similar to case 7	Stationary
Roberts (1969) Case 6 similar to case 7	Stationary
Case 7 similar to case 7	Stationary
Roberts (1969), Case 8	Stationary
Roberts (1969), Case 10	Stationary
Johnson (1969), case 10	Stationary
Corsellis (1973), Case 11	Stationary
Raevuori-Nallinmaa (1950), case 1	Stationary, improvement
Critchley (1957), case 8	Stationary, motor
Roberts (1969), Case 1	Stationary, motor
Johnson (1969), case 6	Stationary, motor and memory

Author, case	Progression Rating
Martland (1928), case 2	NDD-like
Parker (1934), Case 1	Improvement; motor
Parker (1934), Case 2	Improvement; motor
Parker (1934), Case 3	Improvement; motor
Herzog (1938) Case 1	NM
Herzog (1938) Case 2	NM
Herzog (1938) Case 3	NM
Herzog (1938) Case 4	NM
Herzog (1938) Case 5	NM
Herzog (1938) Case 6	NM
Herzog (1938) Case 7	NM
Herzog (1938) Case 8	NM
Herzog (1938) Case 9	NM
Knoll (1938) Case 1	NM
Knoll (1938) Case 2	NM
Knoll (1938) Case 3	NM
Bowman and Blau (1940)	Stationary
Grewel (1941)	Motor
Bourrat and Micolier (1944), case 1	Stationary
Bourrat and Micolier (1944), case 2	NM
McAlpine and Page (1949)	Motor
Critchley (1949) Case A	NM
Critchley (1949) Case B	NM
Critchley (1949) Case C	NM
Critchley (1949) Case D	NM
Critchley (1949) Case E	NM
Critchley (1949) Case F	NM
Critchley (1949) Case G	NM
Raevuori-Nallinmaa (1950), case 1	Stationary, improvement
Raevuori-Nallinmaa (1950), case 2	Motor and memory
Schwarz (1953)	NM
Taylor (1953)	Improvement
Soeder and Arndt (1954), Case 1	NM
Soeder and Arndt (1954), Case 2	NM
Soeder and Arndt (1954), Case 3	NM
Soeder and Arndt (1954), Case 4	NM
Soeder and Arndt (1954), Case 5	NM
Brandenburg and Hallervorden (1954)	NDD-like
Schwarz (1955)	NM
Bergleiter and Jokl (1956)	Complex
Grahmann and Ule (1957), case 1	Complex
Grahmann and Ule (1957), case 2	Complex
Grahmann and Ule (1957), case 3	Complex
Grahmann and Ule (1957), case with pathology	NDD-like
Critchley (1957), case 2	Complex

Author, case	Progression Rating
Critchley (1957), case 3	NM
Critchley (1957), case 4	NM
Critchley (1957), case 8	Stationary, motor
Critchley (1957), case 9	Motor
Critchley (1957), case 10	Motor
Critchley (1957), case 11	Motor
Critchley (1957), Case 12	NM
Critchley (1957), Case 13	NM
Critchley (1957), Case 14	NM
Critchley (1957), Case 15	NM
Critchley (1957), Case 16	NM
Critchley (1957), Case 17	NM
Critchley (1957), case 18	Improvement
Critchley (1957), case 19	Complex
Critchley (1957), Case 20	NM
Critchley (1957), Case 21	NM
Muller (1958), case 1	Complex
Muller (1958), case 2	NM
Muller (1958), case 3	Complex
Neubuerger (1959), case 1	NM
Neubuerger (1959), case 2	NDD-like
Wolowski (1960)	Motor
Courville (1962)	NM
Spillane (1962), case 1	Motor
Spillane (1962), case 2	Motor, complex
Spillane (1962), case 3	NDD-like
Spillane (1962), case 4	NM
Spillane (1962), case 5	NM
Mawdsley and Ferguson (1963), case 1 (? Johnson case 1)	Motor and memory
Mawdsley and Ferguson (1963), case 2	Motor
Mawdsley and Ferguson (1963), case 3	Memory only
Mawdsley and Ferguson (1963), case 4	Memory only
Mawdsley and Ferguson (1963), case 5	Motor
Mawdsley and Ferguson (1963), case 6	Motor
Mawdsley and Ferguson (1963), case 7	Motor and memory
Mawdsley and Ferguson (1963), case 8	Complex
Mawdsley and Ferguson (1963), case 9	NM
Mawdsley and Ferguson (1963), case 10	Memory only, complex
Huszar and Kornvey (1965)	Motor
Burger and Marinoviech	NM
Goralski and Sypniewski (1967)	Improvement, complex
Constantinidis and Tissot (1968)	Complex
Payne (1968), case 1	NM
Payne (1968), case 2	NM
Payne (1968), case 3	NM

Author, case	Progression Rating
Payne (1968), case 4	Stationary
Payne (1968), case 5	NM
Payne (1968), case 6	NM
Betti and Ottino (1969)	NM
Bousseljot (1969), Case 1	Stationary
Bousseljot (1969), Case 2	NM
Bousseljot (1969), Case 3	NM
Bousseljot (1969), Case 4	NM
Bousseljot (1969), Case 5	NM
Bousseljot (1969), Case 6	NM
Bousseljot (1969), Case 7	NM
Bousseljot (1969), Case 8	NM
Roberts (1969), Case 1	Stationary, motor
Roberts (1969), Case 2	Motor
Roberts (1969), Case 3	Motor
Roberts (1969), Case 4	Memory only
Roberts (1969), Case 5	NM
Roberts (1969), Case 6	Motor and Memory
Roberts (1969), Case 7	Complex
Roberts (1969) Case 1 similar to case 7	Stationary
Roberts (1969) Case 2 similar to case 7	Stationary
Roberts (1969) Case 3 similar to case 7	Stationary
Roberts (1969) Case 4 similar to case 7	Stationary
Roberts (1969) Case 5 similar to case 7	Stationary
Roberts (1969) Case 6 similar to case 7	Stationary
Case 7 similar to case 7	Stationary
Roberts (1969), Case 8	Stationary
Roberts (1969), Case 1 similar to case 8	NM
Roberts (1969), Case 2 similar to case 8	NM
Roberts (1969), Case 3 similar to case 8	NM
Roberts (1969), Case 4 similar to case 8	NM
Roberts (1969), Case 5 similar to case 8	NM
Roberts (1969), Case 6 similar to case 8	NM
Roberts (1969), Case 7 similar to case 8	NM
Roberts (1969), Case 9	Motor
Roberts (1969), Case 1 similar to case 9	NM
Roberts (1969), Case 2 similar to case 9	NM
Roberts (1969), Case 3 similar to case 9	NM
Roberts (1969), Case 4 similar to case 9	NM
Roberts (1969), Case 5 similar to case 9	NM
Roberts (1969), Case 10	Stationary
Roberts (1969), Case 1 similar to case 10	NM
Roberts (1969), Case 2 similar to case 10	NM
Roberts (1969), Case 3 similar to case 10	NM
Roberts (1969), Case 4 similar to case 10	NM

Author, case	Progression Rating
Roberts (1969), Case 11	NM
Roberts (1969), Case 1 similar to case 11	NM
Roberts (1969), Case 2 similar to case 11	NM
Roberts (1969), Case 3 similar to case 11	NM
Roberts (1969), Case 1 unrelated to boxing	NM
Roberts (1969), Case 2 unrelated to boxing	Motor and memory
Roberts (1969), Case 3 unrelated to boxing	NM
Roberts (1969), Case 4 unrelated to boxing	NDD-like
Roberts (1969), Case 5 unrelated to boxing	Improvement
Roberts (1969), Case 6 unrelated to boxing	NM
Roberts (1969), Case 7 unrelated to boxing	NM
Roberts (1969), Case 8 unrelated to boxing	NM
Roberts (1969), Case 9 unrelated to boxing	NM
Roberts (1969), Case 10 unrelated to boxing	Memory only
Roberts (1969), Case 11 unrelated to boxing	NM
Johnson (1969), case 1	Complex
Johnson (1969), case 6	Stationary, motor and memory
Johnson (1969), case 10	Stationary
Johnson (1969), case 17	Complex
Corsellis (1973), Case 1	Complex
Corsellis (1973), Case 2	Complex
Corsellis (1973), Case 3	Complex
Corsellis (1973), Case 4	NDD-like
Corsellis (1973), Case 5	Complex
Corsellis (1973), Case 6	Complex
Corsellis (1973), Case 7	Complex
Corsellis (1973), Case 8	Complex
Corsellis (1973), Case 9	Complex
Corsellis (1973), Case 10	NDD-like
Corsellis (1973), Case 11	Stationary
Corsellis (1973), Case 12	NM
Corsellis (1973), Case 13	NDD-like
Corsellis (1973), Case 14	NM
Corsellis (1973), Case 15	NM
Harvey (1974)	Motor
Kaste et al. (1982), Case 1	NM
Kaste et al. (1982), Case 2	NM
Kaste et al. (1982), Case 3	NM
Kaste et al. (1982), Case 4	NM
Kaste et al. (1982), Case 5	NM
Kaste et al. (1982), Case 6	NM
Kaste et al. (1982), Case 7	NM
Kaste et al. (1982), Case 8	NM
Kaste et al. (1982), Case 9	NM
Kaste et al. (1982), Case 10	NM

**Author, case**	**Progression Rating**
Kaste et al. (1982), Case 11	NM
Kaste et al. (1982), Case 12	NM
Kaste et al. (1982), Case 13	NM
Kaste et al. (1982), Case 14	NM
Casson et al. (1984), Case 1	NM
Casson et al. (1984), Case 2	NM
Casson et al. (1984), Case 3	NM
Casson et al. (1984), Case 4	NM
Casson et al. (1984), Case 5	NM
Casson et al. (1984), Case 6	NM
Casson et al. (1984), Case 7	NM
Casson et al. (1984), Case 8	NM
Casson et al. (1984), Case 9	NM
Casson et al. (1984), Case 10	NM
Casson et al. (1984), Case 11	NM
Casson et al. (1984), Case 12	NM
Casson et al. (1984), Case 13	NM
Casson et al. (1984), Case 14	NM
Casson et al. (1984), Case 15	NM
Casson et al. (1984), Case 16	NM
Casson et al. (1984), Case 17	NM
Casson et al. (1984), Case 18	NM
Sabharwal et al. (1987), Case 1	NM
Sabharwal et al. (1987), Case 2	NM
Sabharwal et al. (1987), Case 3	NM
Sabharwal et al. (1987), Case 4	NM
Friedman (1989)	NDD-like
Hof et al. (1992), case 1	NM
Hof et al. (1992), case 2	NM
Jordan et al. (1995)	NDD-like
Geddes et al. (1996)	NM
Jordan et al. (1997), case 1	NDD-like
Jordan et al. (1997), case 2	NM
Jordan et al. (1997), case 3	NM
Jordan et al. (1997), case 4	NM
Jordan et al. (1997), case 5	NM
Jordan et al. (1997), case 6	NM
Jordan et al. (1997), case 7	NM
Jordan et al. (1997), case 8	NM
Jordan et al. (1997), case 9	NM
Jordan et al. (1997), case 10	NM
Jordan et al. (1997), case 11	NM
Jordan et al. (1997), case 12	NM
Jordan et al. (1997), case 13	NM
Jordan et al. (1997), case 14	NM

Author, case	Progression Rating
Jordan et al. (1997), case 15	NM
Jordan et al. (1997), case 16	NM
Jordan et al. (1997), case 17	NM
Jordan et al. (1997), case 18	NM
Jordan et al. (1997), case 19	NM
Jordan et al. (1997), case 20	NM
Jordan et al. (1997), case 21	NM
Jordan et al. (1997), case 22	NM
Jordan et al. (1997), case 23	NM
Jordan et al. (1997), case 24	NM
Jordan et al. (1997), case 25	NM
Jordan et al. (1997), case 26	NM
Jordan et al. (1997), case 27	NM
Jordan et al. (1997), case 28	NM
Jordan et al. (1997), case 29	NM
Jordan et al. (1997), case 30	NM
Geddes et al. (1999)	NM
Drachman (1999)	NDD-like

Table S5. Cases with quotes.

Author, case	Quotes from article	Progression*
Martland (1928), case 2	“Since 1913 his condition has slowly progressed until he now resembles a well-marked case of paralysis agitans.”<br>Condition at retirement not described.	NDD-like
Parker (1934), Case 1	“While in training, his legs seemed to get worse and he found that he tired very readily. He seemed to be fast enough on his feet in the ring at the beginning, but was unable to endure any considerable length of time, because of a sense of fatigue of his lower extremities and a tendency of them to drag. He had given up the idea of fighting.”<br>“From that time up to his admission at the clinic (1934) his condition had changed little.”<br>“The sense of balance was not normal, but slightly better than it had been;”	Improvement; motor
Parker (1934), Case 2	“Walking became increasingly difficult; he noticed that his voice had become nasal, and that articulate speech was becoming more and more indistinct. He continued at the mill about 18 months, when he was discharged because his infirmity had become obvious.” “In this letter he stated he was still able to get around, had not become any worse, and, if anything, there was a slight improvement during the six years that had elapsed since his examination at the clinic.”	Improvement; motor
Parker (1934), Case 3	“Nevertheless, remorselessly his disease advanced, speed and precision were slower in appearing with each bout, and a few days before the patient's first visit to the clinic his career ended in an inglorious fashion.” “A diagnosis was made of lateral sclerosis of the spinal cord, and some doubt was expressed as to the origin of the condition. Syphilis was suspected because of the history, and a course of ant syphilitic treatment was advised on empirical grounds. A very poor prognosis was given, since it was expected that progression of the disease could be expected. The patient was lost track of thereafter, until, for the purpose of the present investigation, he was written to, and in response he reappeared on March 7, 1934, almost exactly eleven years after his first examination. To our surprise we found that time had dealt gently with him. He seemed in no way worse, and beyond acquiring a certain amount of obesity he presented an identically similar picture to that encountered on his first examination, and was, if anything, somewhat improved, discounting the increased weight and lack of training. His disturbance of speech and gait were as conspicuous as before, but he had not deteriorated any more, physically or mentally.”	Improvement; motor
Herzog (1938) Case 1		NM
Herzog (1938) Case 2		NM
Herzog (1938) Case 3		NM
Herzog (1938) Case 4		NM
Herzog (1938) Case 5		NM
Herzog (1938) Case 6		NM
Herzog (1938) Case 7		NM
Herzog (1938) Case 8		NM
Herzog (1938) Case 9		NM
Knoll (1938) Case 1		NM
Knoll (1938) Case 2		NM
Knoll (1938) Case 3		NM
Bowman and Blau (1940)	“A recent report (1 ½ years later) indicated that he was unimproved. His usual indifferent, dull attitude was occasionally disturbed by periods of excitement and assaultiveness but otherwise there were no significant changes.”	Stationary
Grewel (1941)	“In the following four years, a bilateral ptosis developed, and there was a marked tremor, right > left. Changing tremor of the head.”	Motor
Bourrat and Micolier (1944), case 1	“Personally, the patient is concerned because there is no improvement in his condition, despite the fact that he has been in ambulatory and clinical treatment for the last four years.”	Stationary
Bourrat and Micolier (1944), case 2		NM
McAlpine and Page (1949)	February 1948, after losing his temper, his whole body shook and he felt as though he wanted to “get going” with his fists. This quickly subsided but two days later he noticed slight tremor of the left hand, gradually increasing in severity, and, during the next few weeks, spreading up the arm to involve the shoulder and head. Excessive salivation was also noticed during this period, and in March 1948 his speech became slow and indistinct and walking unsteady. All symptoms have been slowly progressive although the tremor was temporarily improved by moderate doses of belladonna. (note: short time interval - January to March 1948)	Motor

Author, case	Quotes from article	Progression*
Critchley (1949) Case A		NM
Critchley (1949) Case B		NM
Critchley (1949) Case C		NM
Critchley (1949) Case D		NM
Critchley (1949) Case E		NM
Critchley (1949) Case F		NM
Critchley (1949) Case G		NM
Raevuori-Nallinmaa (1950), case 1	"During a hospital observation of 5 months there were no changes to speak of in his condition save that the headache grew less. After leaving the hospital he took up manual work later than his former job."	Stationary, improvement
Raevuori-Nallinmaa (1950), case 2	"Memory grown worse." "During 2 years the speech has become indistinct and by degrees grown worse."	Motor and memory
Schwarz (1953)		NM
Taylor (1953)	"Gradual improvement took place over a period of six months with sedation and rehabilitation"	Improvement
Soeder and Arndt (1954), Case 1		NM
Soeder and Arndt (1954), Case 2		NM
Soeder and Arndt (1954), Case 3		NM
Soeder and Arndt (1954), Case 4		NM
Soeder and Arndt (1954), Case 5		NM
Brandenburg and Hallervorden (1954)	Finally, stationary foster care became necessary.<br>Became "soft" but worked as a trademan at retirement; no specific neurological or psychiatric impairment.<br>Progression over years rather than decades.	NDD-like
Schwarz (1955)		NM
Bergleiter and Jokl (1956)	"Following period, absences occurred, and a decrease in intellectual function developed."<br>Symptomatic while boxing: Yes Reflexes were increased on the left side. Despite this continued boxing and was knocked out in the next bout with the following amnestic period. Patient gave up boxing because he felt helpless in the ring.	Complex
Grahmann and Ule (1957), case 1	"A year later, he gives the impression of being demented."<br>Condition at retirement unclear.<br>Bicycle accident	Complex
Grahmann and Ule (1957), case 2	After returning home, his family members were struck by his irritability and fury. He obtained a license as a professional boxer; his career and professional ends after four severe knockout, in a relatively short period of time. After the last knock out he became apathetic, indifferent and slovenly. Could not hold down a job. February 1951 he became excited, pulled the curtains from the windows, destroyed the radio and put his hand into a burning oven.<br><br>Neurological examination reveals severe dementia: Chronic encephalopathy due to boxing is discussed as the cause. Transferred to a psychiatric hospital. Loss of critical faculty, childish euphoria, and marked lack of initiative.<br>PEG: Plump ventricular system and frontal cortical atrophy. In the summer 1955, severe state of agitation, in which he insults and threatens his family. Self-inflicted wound of the right hand.<br><br>Transfer to psychiatric hospital. Here he presented in a catatonic picture. Patient lies in bed grimacing with elevated head. He behaves autistically; from time to time he laughs aloud, without any apparent reason. Pulls out the hairs on his head and then 4 hours sways upper torso back-and-forth. Thoughts obviously incohesive, impossible to get his attention. Sensible conversation not possible, language	Complex

Author, case	Quotes from article	Progression*
	completely incoherent. When asked, why did you destroy the radio?: He replied "it always disturbed me. I had expected dynamite in the radio, because the voices I heard told me that. Then I picked it up and saw that there was no cord connected to it." "Where the voices come from?" "From everywhere. I could not defend myself against them."	
Grahmann and Ule (1957), case 3	Allegedly never knocked out, but sometimes groggy after blows against the head. During the time he boxed, in 1942, friends recognized that his speech became slurred. In 1947 he participated in a series of boxing bouts and took a series of heavy blows against the head, became severely groggy and gave up boxing. The trainer of the boxing club remarked, "they beat him to a pulp." He complains of dizziness, his legs seem insecure and weak, as if they were made of rubber. He complains of headache. Somatic and psychiatric capacity is decreased. Finally he became an invalid. Due to his indistinct babbling speech and his wide leg and swaying gait, he is often believed drunk. The children chased him in the street.<br><br>Since 1951 he has complained of difficulty in swallowing. His wife thinks he has become very irritable and gets excited about minor things.<br>Symptomatic at retirement<br><br>Symptomatic while boxing: Yes - In 1947 he participated in a series of boxing bouts and took a series of heavy blows against the head, became severely groggy and gave up boxing. The trainer of the boxing club remarked, "they beat him to a pulp." He complains of dizziness, his legs seem insecure and weak, as if they were made of rubber. He complains of headache. Somatic and psychiatric capacity is decreased.	Complex
Grahmann and Ule (1957), case with pathology	After 10 years of boxing, he became "soft," he gave up boxing at the age of 25 years and worked as a lock keeper. At 35 years old, he had his first attack of convulsions of the right arm and leg, without consciousness. Repeated attacks occurred, at irregular intervals; they ceased after one year, without treatment. This first his wife noticed the first mental changes and the patient, who is now 36 years old. He was slowed down, uninterested, sitting around idle, cried about nothing and became more forgetful. During this time he perspired noticeably, having taken a corresponding amount of additional fluids. The patient had never been seriously ill, with the exception of a nasal fracture from boxing; he did not smoke and drink.<br>1<br>Admits to admission to a hospital: Robustly built, with coarse features, traumatic saddle nose, deformed ears ("cauliflower ears"). Expression was poor, staring gaze, left corner of his mouth less innervated. Slow, sluggish speech. Patellar tendon reflexes more brisk on the left than the right.<br><br>EEG: Slightly abnormal but with no local disturbances or spikes.<br><br>PEG: External and internal hydrocephalus.<br><br>Mentally: Lack of impulse, retardation, impeded comprehension, memory decreased.<br><br>Could not hold a steady job due to incompetence, discharge from work and unemployable. At this point deterioration was quick; his wife found him "motionless and dull." He was good-natured, in general, but sometimes became irritated and explosive. He required providing for, like a child: Dressing, toilet, did not find his way in the apartment, and moderate eating. Libido and potency completely lost.<br><br>After one year he was once again admitted to a hospital this time in considerably worse condition. Extremely demented, destitute, filthy, lying in bed without movements, an imbecil simper at his lips. He took no notice of the surroundings, interested only in eating. He stopped food eagerly into his mouth, until he was no longer able to close it.<br><br>Personally and locally oriented, but always gave the wrong date. Memory lapses over the past 10 years. Decreased memory, amnestic functions were not as bad as what as one might expect from his desolate condition. The handwriting, still intelligible just two years earlier, was now a narrow, small scribble. He was yielding, willing, good-natured, could not distinguish between left and right. Associated movements of the left arm were missing, short stepping, shuffling gait. Rigid face, on modulated speech.<br>Functional at retirement (worked as a lock keeper; first symptoms of convulsion after 10 years)<br>Died at age 46 with "dementia" according to Boxing: Medical Aspects	NDD-like
Critchley (1957), case 2	"he rapidly deteriorated both medically and professionally"<br>Symptomatic while still competing.<br><br>Symptomatic while boxing: Yes It was noticed by others that he was becoming a little slower, and the speech somewhat thick. On the occasion of his last contest he was knocked out, and headaches thereafter	Complex

Author, case	Quotes from article	Progression*
	became troublesome. Although the patient maintained that he felt well, he was noticeably dysarthric and slow in cerebration.	
Critchley (1957), case 3		NM
Critchley (1957), case 4		NM
Critchley (1957), case 8	"About this time -that is, when aged 27 years-he was rejected at the recruiting office, and he realized then that his sight was poor and that his gait was unsteady. Three months later his hands became tremulous. His symptoms increased up to a point and then remained stationary."	Stationary, motor
Critchley (1957), case 9	"His symptoms consisted of progressively increasing tremor and clumsiness of the right upper limb."	Motor
Critchley (1957), case 10	"In 1948 (aged 24) after losing his temper with his fiance, he started to tremble all over. This subsided except in the left hand, where the tremor persisted with increasing amplitude. After six months it involved the head, and his leg began to drag. His articulation altered."	Motor
Critchley (1957), case 11	A man aged 39, who had been four times schoolboy champion of Great Britain, began to develop tremor of the arm during the war, on which account he was invalided from the Army. His symptoms increased slightly and he showed an impassive, rather mask-like face; marked dysarthria; titubation of the head, with some hypertrophy of the muscles of the neck; incoordination of the arms and legs, more so on the left; with intention tremor and dysdiadochokinesis. Originally of superior intelligence, he later showed both an intellectual falling-off and a curious alteration in personality.	Motor
Critchley (1957), Case 12		NM
Critchley (1957), Case 13		NM
Critchley (1957), Case 14		NM
Critchley (1957), Case 15		NM
Critchley (1957), Case 16		NM
Critchley (1957), Case 17		NM
Critchley (1957), case 18	He seemed to improve after a month's convalescence in Swansea, but, still being unsteady, he went into the National HospitaI, where he was found to be punch-drunk.	Improvement
Critchley (1957), case 19	A heavyweight aged 31 was knocked out "through the ropes" in 1945 and was dazed for the next 24 hours. The right leg was found to be weak immediately after his fight and continued so. Within the day or two following this same fight his right hand began to shake: the left hand started to tremble some six months later. He was hospitalized seven years later, a typical severe punch-drunk.	Complex
Critchley (1957), Case 20		NM
Critchley (1957), Case 21		NM
Muller (1958), case 1	"According to the expert reports," (possible legal case)<br><br>"About 2 to 3 months later, for the first time he had headaches, and noticed an increase slowness which was used repeatedly by his employers as a reason to fire him. Slow development of speech and gait disturbances; clumsiness of hands, progressive social depravation. Several follow-ups since 1953 showed early aging and progressive disintegration of his personality with dementia, and especially an inability of the critical faculties.<br><br>Neurologically he showed spastic gait, coordination disturbances of the upper extremities, dysarthric but not staccato speech. Progression of neurological disturbances in the following years.	Complex
Muller (1958), case 2		NM
Muller (1958), case 3	"The progression of the entire process speaks more in favor of the latter."<br><br>Symptomatic while boxing: Unclear	Complex

Author, case	Quotes from article	Progression*
	Decades: No<br>Co-morbidities: TBI from motorcycle accident<br>Paucity of information: Yes	
Neubuerger (1959), case 1		NM
Neubuerger (1959), case 2	His first known admission to a hospital was at the age of 48, for a cholecystectomy. Neurologic and personality changes were not recorded. His wife stated that during the following year he became forgetful, confused, irritable, and moody. He was examined at the Mayo Clinic, where the following observations were recorded: He was an affable, alert, restless patient, disoriented as to time and place, able to perform only the simplest calculations, and unable to find his way about Rochester unescorted. He showed an ataxic gait, decreased speed of motion in the left hand, increased tendon reflexes, and extensor plantar reflexes on the left. X-rays of the skull and electroencephalogram were interpreted as normal. The impression was "psychotic reaction—the result of organic brain disease—most likely in the nature of a traumatic encephalopathy (punch drunk)." In addition, a diagnosis of mild pulmonary fibrosis and polycythemia (Hgb, 16.8 gm. %; RBC, 5,920,000) was made.<br><br>The patient's condition deteriorated progressively, Several hospitalizations were necessary because of confusion, hyperactivity, loquaciousness, and "nervous breakdown." He died of progressive pulmonary insufficiency, having required oxygen continuously for the last few months of his life. A careful review of all the records indicated that trauma was the only factor that might have played a primary role in the cerebral disorder. His parents had died at advanced ages, of unrelated illnesses. His brother and sister were living, older than the patient, and in good health. The pulmonary insufficiency developed about three years after the onset of the disturbance in mental function, and it<br>did not become severe until 18 months before death.	NDD-like
Wolowski (1960)	"Unsteadiness of gait increased, and he was asked to stop boxing. "<br>"In the last few years he had noticed a decrease in sexual potency, and during the last year he was impotent. According to his wife and close relatives, his illness changed both his character and intelligence."	Motor
Courville (1962)		NM
Spillane (1962), case 1	"Both the unsteadiness and slurring of speech have gradually increased over the years and seem to fluctuate. In the past two years two further symptoms made their appearance."	Motor
Spillane (1962), case 2	His doctor had referred him because of increasing weakness and lack of control of his right leg.<br>Retired at 35. Symptomatic at 40. He recalled a sudden weakness of his right leg while carrying a sack of coal. At first the symptom was intermittent, but over the years it has increased and he has found that he catches the toes of the right foot in the ground as he walks etc etc<br>.During the past three years his gait has been increasingly slow and unsteady, and he has often been thought to be drunk. He is abstemious but has found that alcohol aggravates his ataxia. He works regularly and still feels strong. He has no headache, vertigo, or diplopia. He was not aware of any difficulty with speech, and had no complaints to make suggesting any alteration of personality or intellectual impairment. He has been kept under observation during the past two years and air encephalography was recently repeated. He is still working, although under some difficulty, but he is slower mentally. At times he is anxious or depressed. but on the whole his personality has not significantly altered. Psychometry showed that his level of intelligence was dull normal (I.Q. 81) and that there was significant intellectual deterioration.<br>History suggests radiculopathy – sudden onset of weakness while carrying a sack of coal.	Motor, complex
Spillane (1962), case 3	This man, aged 69, retired from the ring at 32 and was found to be suffering from diabetes six years ago. He had had several hundred fights from the age of 13. For about ten years it had been evident to his friends that his mental faculties were failing. His memory was seriously impaired, there were episodes of gross amnesia and confusion and<br>disorientation. He was childish at times, had little insight, and was content and euphoric. He was nearly always smiling and optimistic. Short journeys often resulted in disorientation. On one occasion, while driving a car, he was involved in an accident and sustained a minor head injury, but was confused and disorientated for several days; he had complete amnesia for the accident itself.<br><br>In 1959 he was admitted to hospital with acute appendicitis, and it was then noted that he could not give a satisfactory history and was confused and disorientated.<br>No indication of disability, followed by progressive deterioration.	NDD-like
Spillane (1962), case 4		NM
Spillane (1962), case 5		NM

Author, case	Quotes from article	Progression*
Mawdsley and Ferguson (1963), case 1 (? Johnson case 1)	“His speech and gait slowly deteriorated.” “His family noted a progressive deterioration in his memory in the past 20 years.”<br>Comment: retired at 32, symptomatic from age 30	Motor and memory
Mawdsley and Ferguson (1963), case 2	“The unsteadiness of his gait slowly worsened. “ “His disability gradually increased though he managed to work as a road sweeper until 10 years ago.”	Motor
Mawdsley and Ferguson (1963), case 3	“At the age of 50 he and his family noticed a deterioration in his memory. This defect was severe for recent events and progressed.”	Memory only
Mawdsley and Ferguson (1963), case 4	“In the past 3 years his wife noticed deterioration in his memory.“	Memory only
Mawdsley and Ferguson (1963), case 5	“When he was about 35 his gait became unsteady and his speech became slurred. The symptoms have progressed.”	Motor
Mawdsley and Ferguson (1963), case 6	At the age of 33 his left arm and hand became weak and unsteady. 5 years ago his wife noticed slurring of his speech and unsteadiness of his gait. His writing slowly deteriorated.<br>Comment: retired at 30	Motor
Mawdsley and Ferguson (1963), case 7	“His unsteadiness of gait and his difficulty with speech slowly worsened. He is now unable to walk unaided. In the past 10 years he and his wife had noted a deterioration in his memory. “	Motor and memory
Mawdsley and Ferguson (1963), case 8	He gave up boxing because of deterioration in his vision. His only complaint was of poor eyesight. He denied any other disability and it was impossible to obtain a detailed history from him. He had always been a heavy beer drinker and was involved in many brawls and had convictions for assault. Because of his violent behavior he was admitted to a mental hospital when he was 40. His wife left him 3 years ago because of his drunkenness and violence. He has not worked for 3 years. Will start unemployed he lived as a tramp, begging drinks and sleeping in public laboratories, until he was admitted to a hospital 18 months ago. During his stay there he made himself a nuisance. He frequently escaped to get drunk and often assaulted his fellow–inmates.<br>Comment: could make a case for no progression. Condition at retirement unclear. “…it was impossible to obtain a detailed history from him.”	Complex*
Mawdsley and Ferguson (1963), case 9	When he was 19 he noticed that his speech, previously precise and clipped, was becoming slurred. This was apparent when he spoke quickly and when he was tired. At that time, too, his memory deteriorated and his handwriting became untidy. In the next two years the symptoms persisted, and he attributed his failure and examinations to the falling off in his memory. Fearing he was becoming punch drunk he stopped boxing regularly, and fought only occasionally in the last 5 years. About the time he stopped boxing completely he began to have almost constant occipital, throbbing headaches, which latterly came on at weekly intervals. He remained conscious of a speech difficulty, particularly on the telephone, and believe that his memory was faulty. His only neurological abnormality was slight but definite slurring that there is dysarthria.	NM
Mawdsley and Ferguson (1963), case 10	His wife noticed a progressive mental impairment from the age of 35. His memory deteriorated, and he lost his last job when he was 42 because he could not remember his duties and was unreliable in keeping to a schedule.”<br>Comment: Retired at 35. Progression in memory loss and complex progression of psychiatric signs (“During the last 10 years of his life he spent most of the day sitting apathetic late at home. He became slovenly in his dress and habits, wearing the same clothes and underclothes for weeks on end unless he was badgered to change. He took no interest in his family's welfare. The lethargy was in striking contrast to the liveliness and alertness described by people who knew him in his 20s.”<br>“When he retired many people taunted him with "punchy".”	Memory only, complex
Huszar and Kornvey (1965)	“According to his statement, the strength of his hand has not weakened, but certain movements were difficult to perform and disturbed by grotesque movements. Trying to move his right hand, he frequently noted a movement of his head to the right. Reduced intensities of complaints, but there was no doubt of the progression of this process. Movements of the head to the right were frequently accompanied by twitches of the facial muscle. Increased perspiration, marked forgetfulness, speech clumsy but he reported that this had been so since childhood.”<br>Comment: “Progression of the process” refers to motor signs. Appears that marked forgetfulness “has been so since childhood.”	Motor
Burger and Marinoviech		NM
Goralski and Sypniewski (1967)	“His delusions (mostly paranoid delusions) have increased.” “His wife confirmed the improvement of his psychiatric status.”	Improvement, complex

Author, case	Quotes from article	Progression*
Constantinidis and Tissot (1968)	"All of the neurological signs have steadily worsened quite rapidly, first between the ages of 25 and 35, then much more slowly. The positive elements, aggressiveness, euphoria, megalomania, interpretativeness of the psychiatric syndrome took center stage around the same time, then faded to give way to deficient signs: memory problems, intellectual deficiency, apragmatism."<br><br>"He was hospitalized twice at the Bel-Air Psychiatric Clinic, at age 33, when his behavioral problems were most marked, and at age 58, 4 months before his death. He was no longer tolerated in an institute where he had been placed when his mother died"	Complex
Payne (1968), case 1		NM
Payne (1968), case 2		NM
Payne (1968), case 3		NM
Payne (1968), case 4	"there was no evidence of intellectual deterioration"	Stationary
Payne (1968), case 5		NM
Payne (1968), case 6		NM
Betti and Ottino (1969)		NM
Bousseljot (1969), Case 1	"Readmission to the hospital from November 5 to December 10, 1965 numbness of the left hand and the left side of the face, motor weakness of the left-sided extremities."<br>"Follow-up (August 1966) findings in general the same."	Stationary
Bousseljot (1969), Case 2		NM
Bousseljot (1969), Case 3		NM
Bousseljot (1969), Case 4		NM
Bousseljot (1969), Case 5		NM
Bousseljot (1969), Case 6		NM
Bousseljot (1969), Case 7		NM
Bousseljot (1969), Case 8		NM
Roberts (1969), Case 1	"These tests, used on his initial admission to the hospital and repeated seven years later, did not show any decline of the scores, nor had his dementia, as judged clinically and by his behavior generally, progressed notably in that time"<br>"the dragging of his left leg had become more apparent as he grew older."	Stationary, motor
Roberts (1969), Case 2	"His condition had remained little changed except that he had become increasingly sensitive to alcohol, small quantities apparently making him very drunk, until the gradual development ten years later of a progressively worsening tremor of his limbs"	Motor
Roberts (1969), Case 3	"Both he and his wife have been aware that his speech, for at least the past ten years and perhaps longer, had been getting "thick and getting thicker""	Motor
Roberts (1969), Case 4	"When, during the course of the examination, and inconstant parkinsonian tremor of his right arm was drawn to his attention, he claimed it had been present only for a few months. He felt that his memory had deteriorated in recent years, so that he had great difficulty with remembering people's names and errands his wife gave him."	Memory only
Roberts (1969), Case 5		NM
Roberts (1969), Case 6	"Since then his speech and gait head progressively deteriorated and his memory, always poor, and he had become so defective that recent years he did not know what day it was."	Motor and Memory
Roberts (1969), Case 7	"His wife had noticed that for many years his speech was slurred when he was excited, but thought that this had become much more obvious since his mild head injury, since when she also noticed that he was unsteady on his feet and tended to drag his left leg"	Complex
Roberts (1969) Case 1 similar to case 7	"There were seven others (sample numbers 4, 14, 29, 67, 133, 169, 216) whose ages range from 38-58 who presented a similar clinical picture of dysarthria and disequilibrium with asymmetrical ataxia as evidence of cerebellar lesions, together with pyramidal lesions and immobile facies, but with little or no overt intellectual defect. One of these had had a longstanding paranoid delusional illness, for which he had undergone psychiatric treatment and another was subject to outbursts of uncontrollable temper requiring	Stationary

Author, case	Quotes from article	Progression*
	sedation. The wife of another described a progressive memory impairment and personality change with marked apathy. All were employed. When independent accounts were available, or the individuals retain insight, there was good evidence that the disabilities had developed during their last years of boxing and had been little changed, or become only very slowly more apparent, as they grew older."	
Roberts (1969) Case 2 similar to case 7	See case 1 similar to case 7	Stationary
Roberts (1969) Case 3 similar to case 7		Stationary
Roberts (1969) Case 4 similar to case 7		Stationary
Roberts (1969) Case 5 similar to case 7		Stationary
Roberts (1969) Case 6 similar to case 7		Stationary
Case 7 similar to case 7		Stationary
Roberts (1969), Case 8	"His wife commented that he was forgetful and agreed that his speech was slurred, but did not think there had been any change in the latter or in his equitable temperament since she had known him a year before he became a professional boxer."	Stationary
Roberts (1969), Case 1 similar to case 8		NM
Roberts (1969), Case 2 similar to case 8		NM
Roberts (1969), Case 3 similar to case 8		NM
Roberts (1969), Case 4 similar to case 8		NM
Roberts (1969), Case 5 similar to case 8		NM
Roberts (1969), Case 6 similar to case 8		NM
Roberts (1969), Case 7 similar to case 8		NM
Roberts (1969), Case 9	"He himself was not aware of this but had noticed that he was unsteady on his feet during his last year's boxing and that this had become worse in recent years so that he often lost his balance."	Motor
Roberts (1969), Case 1 similar to case 9		NM
Roberts (1969), Case 2 similar to case 9		NM
Roberts (1969), Case 3 similar to case 9		NM
Roberts (1969), Case 4 similar to case 9		NM
Roberts (1969), Case 5 similar to case 9		NM
Roberts (1969), Case 10	"She admitted that she had noticed that his speech was slurred about the time he retired from boxing but did not think that there had been any change, for better or worse, since."	Stationary
Roberts (1969), Case 1 similar to case 10		NM
Roberts (1969), Case 2 similar to case 10		NM
Roberts (1969), Case 3 similar to case 10		NM
Roberts (1969), Case 4 similar to case 10		NM

Author, case	Quotes from article	Progression*
Roberts (1969), Case 11		NM
Roberts (1969), Case 1 similar to case 11		NM
Roberts (1969), Case 2 similar to case 11		NM
Roberts (1969), Case 3 similar to case 11		NM
Roberts (1969), Case 1 unrelated to boxing		NM
Roberts (1969), Case 2 unrelated to boxing	"his gait which he has been aware was unsteady since he gave up boxing had become much more so, and his memory had deteriorated."	Motor and memory
Roberts (1969), Case 3 unrelated to boxing		NM
Roberts (1969), Case 4 unrelated to boxing	"Neither he nor his wife had been aware of any neurological disability until he first noticed, at the age of about forty-seven, some difficulty in using his right arm. It was not until five years later that he developed a slowly worsening tremor of his arm which, in the course of a few months, involve all four limbs and was associated with occasional falls, sialorrhea and excessive sweating typical of paralysis agitans. His speech had, since then, become faint and his memory poor."<br>Comment: Considered to be Parkinson's disease by Roberts<br>No symptoms at retirement. "Neither he nor his wife had been aware of any neurological disability until he first noticed, at the age of about forty-seven, some difficulty in using his right arm."	NDD-like
Roberts (1969), Case 5 unrelated to boxing	"He made a complete symptomatic recovery over the course of the next six months."	Improvement
Roberts (1969), Case 6 unrelated to boxing		NM
Roberts (1969), Case 7 unrelated to boxing		NM
Roberts (1969), Case 8 unrelated to boxing		NM
Roberts (1969), Case 9 unrelated to boxing		NM
Roberts (1969), Case 10 unrelated to boxing	"He and his wife thought his memory has been poor since the end of his boxing career and had progressively worsened over the years, so that, for many years, he had to rely entirely on his wife, forgetting, himself, almost anything he was told immediately. Neither he nor his wife were aware of any other neurological disabilities."<br><br>"Examined, his mentation was markedly slow, and his memory was obviously severely defective, not only in recall of the details of his boxing career but of the details of his marriage, past health and occupational history. He was unable to recall the name of the hospital his wife had brought him to and had no idea where it was. On the other hand, he knew the date, and apart from his memory defects, was able to give a coherent account."	Memory only
Roberts (1969), Case 11 unrelated to boxing		NM
Johnson (1969), case 1	"Over the past 20 years there has been a progressive, insidious deterioration of memory and intellect." "Neurological and psychiatric symptoms have progressed. He now presents with severe dementia and is disabled with severe parkinsonian symptoms, although he still lives with his family".	Complex
Johnson (1969), case 6	"A 6o-year-old light-weight, ex-professional boxer retired at 30 years from the ring when he developed severe intention tremor of the right hand, ataxic gait and poor memory for recent events. The condition progressed for five years but has since been relatively stationary."	Stationary, motor and memory
Johnson (1969), case 10	"According to records of his previous neurological examinations these symptoms are not progressive."	Stationary
Johnson (1969), case 17	"He has deteriorated in his social and personal habits." "Although he has deteriorated intellectually and socially over this period he has become placid with the help of phenothiazines"	Complex
Corsellis (1973), Case 1	"He married in his early 20s when he already become a social as well as a boxing success. Soon his life became more hectic and 'he changed completely'. He wenched and drank and gambled heavily. His memory began to fail him. He had three car accidents; in one he suffered severe scalp lacerations and an injury to the right eye, followed by three months in the hospital. His marriage broke up and he drifted	Complex

Author, case	Quotes from article	Progression*
	away from his family, only returning for an occasional embarrassing visit. He had violent outbursts, he was 'knocked out' by only a small amount of alcohol, his behaviour was 'disgusting'. His brother remarked that 'his brain was not functioning – he made mistakes in reckoning'. He could not settle in a job and he became a vagrant. In his 50s his jaw was broken in a brawl. At the age of 62 he was found lying neglected and louse-ridden in the boiler house of a hotel."	
Corsellis (1973), Case 2	"He had boxed from boyhood at first in booths where he often fought several times a day, and later throughout the world as a professional. He was a World Champion for some years and reputedly had more than 700 contests. He retired in his early 30s after a severe battering and was described at that time as 'a little old man…playing the part of an animated punching bag….By the age of 50 he staggered slightly when he walked, and his speech was slow and slurred – 'In fact you'd think he was drunk but he never touched a drop'. During the following decade he became childlike, often wanting to be cuddled and reassured. He suffered two head injuries which were not severe but they may have aggravated the deterioration in memory and behavior; while recovering from the second he was found to be mildly diabetic…When he was 67 he developed an acute appendicitis and was noticed to be confused, incontinent of urine, and disorientated; his memory was severely impaired. His manner was still childish; he would sulk when left alone and would smile happily when approached. He retained some idea of his boxing success but had forgotten the details. He spoke indistinctly. He was markedly ataxic, walking on a wide base and often stumbling. He could not stand on one leg and heel-toe walking led him to fall to the right. He had nystagmus to the right and a tremor of his upper limbs. Muscle power, tendon reflexes, pupillary responses, and blood pressure were all normal…The cerebrospinal fluid was normal and serological tests for syphilis were negative. A lumbar air encephalogram showed marked symmetrical dilatation of the lateral ventricles and a well-outlined septal cavum. Cerebral atrophy and the punch-drunk syndrome were diagnosed…<br>He died demented and doubly incontinent, in a psychiatric hospital at the age of 77. Death was attributed to bronchopneumonia as a consequence of organic dementia."	Complex
Corsellis (1973), Case 3	"This boxer's wife was interviewed. He was born abroad and came to England as a young child. He started boxing at 16 and fought about 300 professional contests during the next 13 years, winning an area championship and traveling abroad. He suffered a neck injury (unrelated to boxing) when about 26 and boxed less after this, becoming a manager when approaching 30. His wife met him shortly after this and she recalled that "he had already begun to get a bit muddled". By the age of 36 he would often fall over backwards. He began to suspect his mother of theft and his wife of infidelity. He was seen in hospital when age and 44 because of back pain. Osteoarthritis of his cervical vertebrae was found on x-ray; a rigid gait was noted, both arms were slightly spastic; early parkinsonism was queried. At 50, slurring of the speech was mentioned and the term "punch drunk" was used. 6 years later a neurological report included an extrapyramidal tremor of both hands and ataxia of both legs. There was a slight dysarthria. An air cephalogram shortly afterwards showed enlarged lateral ventricles with a septal cavum. A further report, when he was 61, stated that his unsteadiness and shaking hands have become worse. He was "dribbling a bit", he had parkinsonian facies, his speech was difficult to understand, and his memory was poor. His wife recalled that he had a bad temper, the saying "the scenes were awful", "he raved at night" but was "so kind and nice" afterwards. It was concluded in the hospital that he had "diffuse degenerative brain disease affecting extrapyramidal and pyramidal systems as well as causing mental impairment" He could not talk or swallow properly; he had several falls and after the last one he died from bronchitis and bronchopneumonia when aged 63. He was said to have drunk moderately at times and attended an attack of pancreatitis when age 55 was attributed to alcohol. He appears to have given up the drink since then."	Complex
Corsellis (1973), Case 4	"When he was 57 he attended hospital for a backache and was found to have a spastic gait and slurred speech, both of which were recorded as having been present for some years. At the age of 64 he was re-investigated in a neurosurgical unit. A history of progressive unsteadiness on his feet and mental deterioration with impairment of memory were described by his wife. He lost all sense of date and time and did not recognize his relatives. His speech was dysarthric, he had a wide-based ataxic gait, and Romberg's sign was positive. He was considered mildly demented."	NDD-like*
Corsellis (1973), Case 5	"He was always aggressive and spiteful and was so unpopular with the crowds that they liked to see him beaten.' He never refused a challenge, regardless of weight, and often took 'terrible punishment' to the head. He sometimes boxed in a trance. He drank excessively in his 20s. Toward the end of his career at the age of about 30 he was no longer able to look after his personal affairs. He became enuretic when about 35 and eventually had to be cared for in a home. He was noted to be 'irascible, forgetful, but clean and tidy and quite nimble on his feet'. When 60 years old he developed a right sided facial weakness and a right homonymous hemianopsia. In the same year, and a few months before he died, he was transferred to a psychiatric hospital. Death was attributed to bronchopneumonia and cardiac ischaemia."	Complex
Corsellis (1973), Case 6	"He was fit until the age of 32 when his legs 'began to give way', he developed a 'hopping-dancing walk', and his speech became slurred. His memory was good. He retired from boxing six years later and became a referee and a promoter. Eventually he took any job he could get. His two marriages broke up, largely because of his violent nature. When 65 years old he went to live with his son but aggressive outbursts led	Complex*

Author, case	Quotes from article	Progression*
	to his removal to an old people's home. He became more subdued but he still had the occasional scuffle and one of these brought him to a psychiatric hospital."	
Corsellis (1973), Case 7	"He retired from the ring after a particularly damaging fight, and started to teach physical training and boxing and boarding schools. He gave this up after a few years at a time when he was drinking heavily and beginning to become moody and violent. A few years later he lost a job in a printing works for hiding away to sleep and he seems not to have worked again. When about 54 he began to be "very unsteady on his feet" and kept falling out. He had spells of "going within himself" followed by aggressive attacks on his wife and his home. He complained of violent headaches; his sexual demands gave his wife a little rest. He wandered, out, half dressed, at night and would import Toone for money. He had "glassy looking eyes"; he was doubly incontinent at times. He spoke normally and had no tremor; he could not be left alone. When 59 he was admitted to a psychiatric hospital and found to be grossly demented."	Complex
Corsellis (1973), Case 8	"He began to go downhill when he was 60 years old. He then lost consciousness for about 2 hours and on recovery he was ataxic and had weakness of his left side. Several similar episodes occurred during the next 10 years; between them he remained ataxic with a tendency to fall. He had a tremor and could not tie a bow. The last attack affected his right side, and his intellect and behavior then deteriorated. He became violent towards his wife but would afterwards apologize. He burnt all his newspaper cuttings and then asked for them back. He would try to go unclothed into the street. He died at home age of 71."	Complex
Corsellis (1973), Case 9	"On this return to England, when aged 31, his brother noticed that there was a tremor in his left hand and that his speech was slurred and hoarse. The hoarseness had followed a blow to the throat…He started drinking heavily and drifted from job to job. He tried to kill himself when aged 65 and was admitted to a psychiatric hospital. A Parkinsonian facies was noted, with tremor and rigidity of the left upper limb. The left vocal cords were markedly thickened and he was dysarthric. The blood pressure ranged around 210/130, and he was considered to be demented possibly as a result of cerebral trauma."	Complex*
Corsellis (1973), Case 10	"She first noticed that his memory was poor when, age about 40, he was serving in her shop. Later he worked as a labourer, and became unemployed for 5 years after the loss of an eye (unrelated to boxing). He returned to work as a road sweeper. He and his wife at drinking bouts and he was known in his locality as a violent man, although "he had been a good husband". At the age of 60 he developed a left hemiparesis and was admitted to a mental hospital 3 years later. He was disoriented and had a marked loss of recent memory. He was paranoid and deluded, and became confused and aggressive. The Kahn and Price's precipitation reactions were negative. The diagnosis was of cerebral arteriosclerosis and organic dementia, probably posttraumatic in origin. The term "punch drunk" was used. He gradually deteriorated over the next 4 years and died at age 67."	NDD-like
Corsellis (1973), Case 11	"During the last 6 years of his life he remained bright and alert and worked regularly as a road sweeper." Comment: Deterioration over 3 months prior to death interpreted as agonal.	Stationary
Corsellis (1973), Case 12		NM
Corsellis (1973), Case 13	"He worked as a mile roundsman, bus conductor, and later as a machine operator. When he could no longer manage this, the firm transferred him to laboring and caretaking. After two years he was given notice without warning. He wandered away bemused and had to be taken to a mental hospital. He was then 53. On admission he was mildly confused and complained of blackouts. His wife, from whom he was separated, said that his personality had been changing for the worse. A brief neurological report confirmed this and mentioned a deterioration of habits. He could not recall his address or the date. No incoordination was found a 'little dyspraxia' was queried. During the next year his memory deteriorated further, he was completely disorientated and needed help with dressing. These features were emphasized in a detailed neurological investigation. In addition his speech was slurred, and like his movements was slow and tremulous. He could not undress himself. An air encephalogram revealed generalized ventricular dilatation. All other investigations, including serological tests for syphilis, were negative and it was concluded that 'the cortical atrophy and dementia may be related to his previous experiences as a boxer…The deterioration continued. He became incontinent, paranoid, and aggressive. He ate off the table with his fingers; a cigarette packet he called a flower; he undid buttons when asked to put his tongue out. He continued to have blackouts and a few days after a convulsion he died from bronchopneumonia, aged 57 years. "	NDD-like
Corsellis (1973), Case 14		NM
Corsellis (1973), Case 15		NM
Harvey (1974)	"his gait and speech deteriorated."	Motor
Kaste et al. (1982), Case 1		NM
Kaste et al. (1982), Case 2		NM

Author, case	Quotes from article	Progression*
Kaste et al. (1982), Case 3		NM
Kaste et al. (1982), Case 4		NM
Kaste et al. (1982), Case 5		NM
Kaste et al. (1982), Case 6		NM
Kaste et al. (1982), Case 7		NM
Kaste et al. (1982), Case 8		NM
Kaste et al. (1982), Case 9		NM
Kaste et al. (1982), Case 10		NM
Kaste et al. (1982), Case 11		NM
Kaste et al. (1982), Case 12		NM
Kaste et al. (1982), Case 13		NM
Kaste et al. (1982), Case 14		NM
Casson et al. (1984), Case 1		NM
Casson et al. (1984), Case 2		NM
Casson et al. (1984), Case 3		NM
Casson et al. (1984), Case 4		NM
Casson et al. (1984), Case 5		NM
Casson et al. (1984), Case 6		NM
Casson et al. (1984), Case 7		NM
Casson et al. (1984), Case 8		NM
Casson et al. (1984), Case 9		NM
Casson et al. (1984), Case 10		NM
Casson et al. (1984), Case 11		NM
Casson et al. (1984), Case 12		NM
Casson et al. (1984), Case 13		NM
Casson et al. (1984), Case 14		NM
Casson et al. (1984), Case 15		NM
Casson et al. (1984), Case 16		NM

Author, case	Quotes from article	Progression*
Casson et al. (1984), Case 17		NM
Casson et al. (1984), Case 18		NM
Sabharwal et al. (1987), Case 1		NM
Sabharwal et al. (1987), Case 2		NM
Sabharwal et al. (1987), Case 3		NM
Sabharwal et al. (1987), Case 4		NM
Friedman (1989)	"30 years after he stopped boxing, this patient had a progressive syndrome of mild dementia, akinesia, rigidity, upper motor neuron signs, postural instability, and a slow, slurred hypophonic dysarthria." The vignette implies relatively neurological preservation at retirement, by noting progressive symptoms 30 years after retirement. "This 55-year-old man was noted by others to be "slowing down" over the past 3 years, though he himself was unaware of it."	NDD-like
Hof et al. (1992), case 1		NM
Hof et al. (1992), case 2		NM
Jordan et al. (1995)	"A neurological syndrome of progressive cognitive decline began 37 years after his last bout (10 years antemortem), and an acute right hemiplegia developed 8 years antemortem, following which he no longer recognized his family. Six weeks antemortem, he suffered a fall and died following two successive left parietooccipital cerebral hemorrhages."	NDD-like
Geddes et al. (1996)		NM
Jordan et al. (1997), case 1	"Severe dementia" in a 72 year old former boxer.	NDD-like*
Jordan et al. (1997), case 2		NM
Jordan et al. (1997), case 3		NM
Jordan et al. (1997), case 4		NM
Jordan et al. (1997), case 5		NM
Jordan et al. (1997), case 6		NM
Jordan et al. (1997), case 7		NM
Jordan et al. (1997), case 8		NM
Jordan et al. (1997), case 9		NM
Jordan et al. (1997), case 10		NM
Jordan et al. (1997), case 11		NM
Jordan et al. (1997), case 12		NM
Jordan et al. (1997), case 13		NM
Jordan et al. (1997), case 14		NM
Jordan et al. (1997), case 15		NM

Author, case	Quotes from article	Progression*
Jordan et al. (1997), case 16		NM
Jordan et al. (1997), case 17		NM
Jordan et al. (1997), case 18		NM
Jordan et al. (1997), case 19		NM
Jordan et al. (1997), case 20		NM
Jordan et al. (1997), case 21		NM
Jordan et al. (1997), case 22		NM
Jordan et al. (1997), case 23		NM
Jordan et al. (1997), case 24		NM
Jordan et al. (1997), case 25		NM
Jordan et al. (1997), case 26		NM
Jordan et al. (1997), case 27		NM
Jordan et al. (1997), case 28		NM
Jordan et al. (1997), case 29		NM
Jordan et al. (1997), case 30		NM
Geddes et al. (1999)		NM
Drachman (1999)	"A 67-year-old, right-handed man was admitted to the hospital because of increasing dementia…Three years before admission, the patient's cognitive function began to decline. One year later, a neurologist diagnosed progressive dementia with parkinsonism…During the two months before admission, the patient became agitated, and trazodone was prescribed. His family noted transient periods when he stared into space and did not respond to his surroundings. He fell several times, with apparent loss of consciousness but no obvious injury…No apparent deficits at retirement from boxing. "The patient had boxed professionally for 10 years in over 100 bouts. He drank alcohol excessively in his youth but had been sober for several decades. There was no family history of psychiatric problems. A sibling had died 10 years earlier of probable amyotrophic lateral sclerosis."	NDD-like

*Stationary – evidence in the record of stable disease
Improvement – evidence in the record of some clinical or neurological problem may have improved over time
Motor – description of progressive disease limited to motor signs or symptoms
Memory – description of progressive disease limited to memory function
Motor and memory - description of progressive disease limited to motor and memory function
Complex – progressive deterioration occurred over a period of decades and involved co-morbidities; in some cases coded as complex, progression was alluded to but contained a paucity of information, and/or case description of progression
NDD-like = description of disease progression consistent with or similar to canonical neurodegenerative disease

Table S6. Boxing history, clinical features, and neuropathology in boxers from Corsellis et al. 1973.

													Corsellis et al.						
#	Age when started boxing	Duration of boxing (years)	Estimated no. of fights	Age at symptom onset	Disease duration (years)	Presenting symptoms & disease progression	Progressive dementia+	Behavioural changes	Mood changes	Motor impairment	Age at death	Final Clinical diagnosis	SF	EV/ A	CTS	NFT	Revised neuropath. diagnosis	ARTAG: Subcortical	ARTAG: Mediobasal
1*	11	14	400	25	38	Dramatic personality changes towards end of his successful boxing career in his mid-20's, excessive alcohol consumption, gambled heavily. In his 30's, episodic memory impairment, violent outbursts, frontal disinhibition, self-neglect. Advanced dementia, dysarthria, unsteady gait in last 3 years.	Yes (Memory impairment in his 30's)	Lifestyle indulgences, excessive alcohol intake, gambled heavily, reckless generosity, social inappropriateness, frontal disinhibition, 'disgusting behaviour', multiple RTAs, social isolation, lived in destitute.	Violent outbursts (30's)	Unsteady gait in his 60's, 'could hardly walk', dysarthria. Extensor plantar response.	63	Punch drunk	Yes	Yes	Yes	+++	CTE	Present	Present
2*^	13	19	700	55	22	Childlike behavioural change, followed by memory impairment in late 50's, disorientation, then cerebellar signs and hand tremor aged 67, headache, and advanced dementia aged 71	Yes (behavioural change followed by memory impairment, intermittent disorientation to place, lacked insight. In late 60's, incontinence)	Childish (aged 55)	Euphoria (50's)	Cerebellar signs with gaze-evoked nystagmus, dysarthria, dysmetria, dysdiadochokinesia, unable to tandem walk, wide-based gait (50's), hand tremor (60's)	77	Punch drunk	Yes	Yes	Yes	+++	CTE	Present	-
3*	16	10	270	36	27	Impairment of episodic memory, 'get muddled', paranoid delusions, irritability, aggression, frontal features, followed by dysarthria, parkinsonism, gait impairment and falls.	Yes (progressive memory impairment, confusion, 'head never felt clear')	Paranoid delusions (theft, infidelity), hypersexuality, physical violence, aggression, rage, hoarding, swearing	Irritability	Parkinsonism (drooling, hypomimia, dysarthria, rigidity, shuffling gait, pill-rolling tremor, marked bradykinesia; aged 44), ataxia with dysmetria, dysdiadochokinesia (aged 56), pseudobulbar palsy, brisk jaw jerk, wasting of small hand muscles, fasciculation in triceps, deltoid.	62	Punch drunk	Yes	Yes	Yes	+++	CTE	-	Present
4	15	25	600	64	5	Progressive unsteadiness, dysarthria, mild cognitive impairment, followed by hypomimia and leg tremor (aged 64). Background of dysarthria, hand tremor, spastic wide-based gait from mid-30's without deterioration.	Yes (Mild cognitive impairment with disorientation in time and place)	Aggression	Irritability	Hypomimia, tremor & rigidity of limbs, shuffling gait (aged 69)	68	Punch drunk	Yes	Yes	Yes	++	Progressive supranuclear palsy/ ARTAG	Present	Present
6*	13	25	500	32	51	Gait impairment, followed by dysarthria, rage and aggression, violence since his 30's, progressive dementia, paranoid in his 60's, and parkinsonism in last 3 years of life	Yes (significant dementia with predominant frontal behavioural changes in his late 60's, incontinence in his 80's)	Aggressive, rage, violent, hypersexuality, impulsivity, explosivity, paranoid delusions	Emotional lability	Hypomimia, slow shuffling gait in his 80's	83	Dementia	Yes	Yes	Yes	++	CTE	Present	Present
7*	13 (According to official record)	19	400	50	12	Impairment of episodic memory, frontal lobe features, aggressive behaviours, severe headache	Yes (progressive memory impairment and executive dysfunction with predominant frontal features. Aged 59, advanced dementia)	Disinhibition, hypersexuality, aggressive, excessive alcohol consumption	Irritability, mood swing, apathy	Ataxia and cerebellar signs (aged 54)	62	Pre-senile dementia	Yes	Yes	No	+++	CTE	-	Present
8*	17	23	565	60	11	Repeated episodes of LOC followed by transient ataxia, left hemiparesis, persistent action hand tremor, then	Yes (memory impairment in last few years)	Childish, disinhibition (go into the street unclothed), irrational acts, physical violence	-	Ataxia (aged 60) and tendency to fall, tremor (60's)	71	Punch drunk TIA	Yes	Yes	No	++	CTE	Present	Present

													Corsellis et al.						
**#**	**Age when started boxing**	**Duration of boxing (years)**	**Estimated no. of fights**	**Age at symptom onset**	**Disease duration (years)**	**Presenting symptoms & disease progression**	**Progressive dementia+**	**Behavioural changes**	**Mood changes**	**Motor impairment**	**Age at death**	**Final Clinical diagnosis**	**SF**	**EV/ A**	**CTS**	**NFT**	**Revised neuropath. diagnosis**	**ARTAG: Subcortical**	**ARTAG: Mediobasal**
						childish behavior, memory impairment, disinhibition, violence													
9	19	12	NK	61	11	Memory impairment, violent outburst, followed by asymmetrical parkinsonism	Yes (since early 60's, diagnosed with dementia aged 66, and spent last 7 years in a psychiatric hospital)	Violent outbursts	Suicide attempt (aged 65)	Asymmetrical parkinsonism with left rest hand tremor, tongue tremor, cogwheel rigidity and hypomimia	72	Punch drunk, pre-senile dementia & parkinsonism	Yes	Slightly	Yes	++	ARTAG/ Lewy body dementia	Present	Present
10*	NK	NK	NK	40	27	Memory impairment	Yes (since aged 60, marked loss of recent memory, disorientated, spent last 4 years in a psychiatric hospital)	Paranoid delusion (aged 60), Always known as a violent man	-	-	67	Punch drunk	Yes	Yes	Yes	++	CTE/ Alzheimer's disease	-	Present
11	NK	NK	NK	NA	NA	(Asymptomatic)	-	-	-	-	67	Neurosyphilis (normal CSF serology post-treatment), otherwise active & well	Yes	Yes	No	+	ARTAG	-	Present
12	NK	NK	NK	NA	NA	(Asymptomatic)	-	-	-	-	91	Registered blind, otherwise active & well	No	Slightly	No	+	No diagnosis	-	-
13	Teens	30	NK	49	7	Cognitive impairment with slowing of speech and gait, difficulty operating machinery at work, followed by neglect of personal appearance, aggressive behavior, wandering, disorientation and dysphasia. Aged 54, no speech, did not recognize his family, no speech output, bedridden.	Yes (executive dysfunction, dressing dyspraxia, dysphasia, memory impairment)	Paranoid, aggressive, violent	Emotional lability	Generalized slowing & gait impairment (aged 49)	56	Punch drunk	NK	Yes	Yes	+++	Alzheimer's disease	-	-
14	18	28	NK	NA	NA	(Asymptomatic)	-	-	-	-	61	Healthy until fatal subarachnoid haemorrhage	NK	-	No	+	ARTAG	-	Present
15	NK	NK	NK	NA	NA	(Asymptomatic)	-	-	-	-	58	Healthy until fatal RTA	NK	-	Yes	0	No Diagnosis	-	-

Note: Brain tissue for case #5 was not available for examination.
*: Cases with path-confirmed CTE applying the most recent NINDs criteria (McKee, Cairns, et al., 2016)
^This case (Case 2 in the Corsellis' series (Corsellis et al., 1973)) corresponds to Case 3 reported in another historical boxer series (Spillane, 1962)
-: Absent, LOC: Loss of consciousness, NA: Not applicable, NK: Not known, RAF: Royal Air Force, RHI: Repetitive head impacts, RTA: Road traffic accidents
+Progressive dementing illness accompanied by symptoms of memory impairment, executive dysfunction, disorientation, aphasia, visuospatial impairment
ARTAG Subcortical=ARTAG: Subcortical white matter (patchy); ARTAG Mediobasal=ARTAG: Mediobasal regions (subependymal, periventricular, perivascular)
Pathological features were extracted from Table 5 of Corseills et al. (1973), with SF=Septum Fenestration, CTS=Cerebellum Tonsillar Scarring, and NFT=Neurofibrillary Tangles in the Cerebral Cortex, with 0=No Change, +=Slight Change, ++=Moderate, and +++=Severe. EV/A=Enlarged ventricles and/or cortical atrophy. This column of information was derived from reading the sections in Corselllis et al. (1973) entitled "Naked-Eye Appearance of Brain" for each case study

The content for this table was derived from the online supplementary Tables 1, 5, and 6 from Goldfinger et al., 2018.
Goldfinger MH, Ling H, Tilley BS, Liu AKL, Davey K, Holton JL, Revesz T, and Gentleman SM. (2018). The aftermath of boxing revisited: identifying chronic traumatic encephalopathy pathology in the original Corsellis boxer series. Acta Neuropathologia 136:973-974.

Some of the content was also derived from Corsellis et al. 1973.
Corsellis JA, Bruton CJ, Freeman-Browne D (1973). The aftermath of boxing. Psychol Med 3:270–303.
